# Pitfalls When Determining HNA-1 Genotypes and Finding Novel Alleles

**DOI:** 10.3390/ijms25169127

**Published:** 2024-08-22

**Authors:** Kirstine Kløve-Mogensen, Tom Browne, Thure Mors Haunstrup, Rudi Steffensen

**Affiliations:** 1 Department of Clinical Immunology, Aalborg University Hospital, 9000 Aalborg, Denmark; t.haunstrup@rn.dk (T.M.H.); rns@rn.dk (R.S.); 2 Service Development Laboratory, NHS Blood and Transplant, London NW9 5BG, UK; tom.browne@nhsbt.nhs.uk; 3 Department of Clinical Medicine, Aalborg University, 9000 Aalborg, Denmark; 4 Department of Clinical Medicine, Aarhus University, 8000 Aarhus, Denmark

**Keywords:** HNA-1, FCGR3B, FCGR3A, PCR, genotyping, long-read sequencing, nanopore, copy number variation

## Abstract

Genetic variation in the *FCGR3B* gene is responsible for different variants of human neutrophil antigen 1 (HNA-1). Laboratory techniques currently utilized for routine HNA-1 genotyping, predominantly PCR-sequence-specific primer (PCR-SSP) and PCR-sequence-based typing (PCR-SBT), lack specificity for *FCGR3B*. This study compares the capabilities and limitations of existing technologies including an in-house TaqMan PCR, a commercial PCR-SSP test, PCR-SBT and multiplex ligation-dependent probe amplification (MLPA) with those of a long-read nanopore sequencing assay. Testing was performed with both related and unrelated Danish samples with different copy numbers and/or rare alleles. Long-read nanopore sequencing was validated by blind testing of ten English samples. The results showed that *FCGR3B* copy numbers correlate with a dose-dependent distribution of alleles that complicates genotyping by TaqMan PCR, PCR-SSP and PCR-SBT, due to co-amplification of the homologous *FCGR3A* gene. MLPA can correctly quantify the dose-dependent distribution but not detect novel variants. Long-read nanopore sequencing showed high specificity for *FCGR3B* and was able to detect dosage-dependent distribution, and rare and novel variants that were previously not described. Current HNA-1 genotyping methods cannot produce unambiguous allele-level results, whereas long-read nanopore sequencing has shown the potential to resolve observed ambiguities, identify new HNA-1 variants and allow definitive allele assignment.

## 1. Introduction

The human neutrophil antigen 1 (HNA-1) is the allotypic form of glycoprotein Fc gamma receptor IIIb (FcγRIIIb, CD16b) expressed on the surface of human neutrophil granulocytes that plays an important role in allo- and autoimmunity [1]. Each neutrophil cell carries between 100,000 and 300,000 copies of FcγRIIIb on the neutrophil membrane, and the density correlates with the number of copies of the coding gene, *FCGR3B* [2,3]. HNA-1 variants play a central role in known clinical complications, such as neonatal alloimmune neutropenia, autoimmune neutropenia and transfusion-related acute lung injury (TRALI) [4,5]. Known HNA-1 variants are a result of six single nucleotide polymorphisms (SNPs) (c.108G/C, c.114C/T, c.194A/G, c.233C/A, c.244G/A and c.316G/A) in exon 3 of *FCGR3B* that induce five amino acid (aa) changes: p.Arg36Ser, p.Asn65Ser, p.Ala78Asp, p.Asp82Asn and p.Val106Ile [6,7,8], as shown in Table 1. According to the ISBT nomenclature, there are five *FCGR3B* alleles (FCGR3B*01-*05), two subvariants and one FCGR3B*null allele [9]. FCGR3B*01 encodes HNA-1a and differs in five nucleotides (c.108G, c.144C, c.194A, c.244G and c.316G), from FCGR3B*02 (c.108C, c.114T, c.194G, c.244A and c.316A)*,* which encodes HNA-1b. The distribution of the two alleles varies between populations, with a frequency for FCGR3B*01 of 58% in Caucasians and 91% in Taiwanese individuals and a frequency for FCGR3B*02 of 88% in Caucasians and 54% in Taiwanese individuals [10]. FCGR3B*03, the allele encoding HNA-1c, differs only at one aa (c.233A, p.78Asp) from HNA-1b [11]. This allele is rare in Europeans, where it is reported to be carried by 5% of German individuals and less than 1% of Danish individuals [12,13]. This allele is missing in the Chinese population, but in Africans, its frequency is approximately 22–39% [13,14]. In Europeans, FCGR3B*03 is in strong linkage disequilibrium with higher copy numbers (CNs) of the *FCGR3B* gene, but this is not observed for Africans [15]. Many Europeans are positive for FCGR3B*03, therefore, exhibit three or more HNA-1 antigens [13]. However, more CNs are not only detected among carriers of FCGR3B*03; among Danes, several combinations of FCGR3B*01 and FCGR3B*02 with higher CNs exist [12]. Gene duplication is the counterpart of gene deletion, and FCGR3B*null alleles are more frequently observed in populations with higher CNs [10,16]. FcγIIIb deficiency (completely lacking *FCGR3B* on both chromosomes) is observed in approximately 1 per 1,000 Europeans and 1 per 100 Africans/African Americans [17,18]. These individuals can be immunized and produce anti-FcγRIIIb isoantibodies [19]. The fourth HNA-1 allele, FCGR3B*04, has one aa different from that of FCGR3B*01 (c.316A, p.106Val) but encodes a protein with unaltered reactivity compared to that of FCGR3B*01 [20,21]. In Caucasians, the FCGR3B*03 was first reported to be segregated with FCGR3B*01 [22], but later studies and newer technologies have shown that it is FCGR3B*04 that segregates with FCGR3B*03 [13]. FCGR3B*05 is characterized as an HNA-1b allele with an aa change (c.244G, p.82Asp) and exhibits reduced reactivity with wild-type HNA-1b antibodies [20,21,23]. In addition to the five confirmed alleles, other variants have been observed but not fully confirmed [23,24,25,26].

Routine laboratory testing of HNA-1 alleles is inferred from the allelic genotype according to the current International Society of Blood Transfusion (ISBT) HNA nomenclature [9]. The ISBT nomenclature defines six SNPs all located in exon 3 of the *FCGR3B* gene that correlate to five defined alleles (FCGR3B*01-*05). The missense mutations responsible for the confirmed alleles are the following SNPs: c.108G/C (rs200688856), c.114C/T (rs527909462), c.194A/G (rs448740), c.233C/A (rs5030738), c.244G/A (rs147574249) and c.316G/A (rs2290834), as shown in Table 1. Polymerase chain reaction with sequence-specific primers (PCR-SSP) has been the most frequently used approach [14,27]; however, the high degree of homology between *FCGR3B* and another FcγR gene, *FCGR3A,* complicates genotyping of *FCGR3B* alleles. The complexity of the *FCGR2/3* locus is further increased by recurrent events of nonallelic homologous recombination, which have given rise to both copy number variation (CNV) and chimeric genes [28,29]. Five copy number regions (CNRs), subjected to either deletion or duplication, have been defined in the *FCGR2/3* locus [3,15]. The most common are CNR1, which encompasses *FCGR2C* (CD32c) and *FCGR3B* (CD16b) and is in most cases coupled to higher and lower CNs of *FCGR3B.* CNs in *FCGR3B* are subject to ethnic variation and vary between zero and seven copies. Genotyping is further complicated with CNVs not only in *FCGR3B* but also in *FCGR3A*, which can vary from 0–4 copies [3]. The CNV of *FCGR3B* is a risk factor for several diseases, including glomerulonephritis, systemic lupus erythematosus, rheumatoid arthritis, sarcoidosis and autoimmune neutropenia [12,30,31,32,33], where the associations are connected to fewer copies or the absence of copies.

The CNV of *FCGR3B* has been reported to influence receptor density at the cell surface [2,34,35], but the dosages of the different HNA-1 alleles as a result of CNV are rarely explored. Autoimmune neutropenia and antibody-associated systemic vasculitis are caused by reduced phagocytosis and are associated with HNA-1a [36,37] and IgG antibody specificity [38]. Systemic lupus erythematosus is known to be associated with increased phagocytosis, which is linked to the HNA-1b genetic variant [38,39,40]. For autoimmune neutropenia, we previously combined CNs and HNA-1 alleles and found that hemizygous HNA-1a was a disease risk [12]. Combining knowledge of CNs, hence the receptor density of the surface, and the types of HNA-1 alleles might help in understanding the role of FcγIIIb in different diseases.

Approximately 12.2% of Danish Blood Donors have CNs alternating from two, 0.2% are nullizygous, 3.8% are hemizygous, 87.8% have two copies, and 8.2% have three or more copies [41]. We previously found one nullizygous individual in the Danish cohort (0.2%), confirming its presence in the Danish population, and the frequency is not expected to change from that of other European populations, which is estimated to be 1/1000 [17]. We also observed that among all tested patients, a higher CN of *FCGR3B* was always followed by a higher CN of *FCGR2C*, as expected for CNR1 [42]. Danish CN frequencies reflect other European studies where the frequency of <2 copies was found to be approximately 6.2–6.6% and >2 copies 9.9–10.9%. However, Europeans seem to be the population with the least CNV, and the frequency in Asians ranges from 6.3–10.2% for <2 copies and 13.9–34.4% for >2 copies [15,43,44], while in Africans <2 copies range from 4.7–18.2% and 6.6–25.8% for >2 copies [15,43,44,45]. The highest frequencies seem to be among South Americans, where the frequency ranges from 4.2–39.3% for <2 copies and 14.3–48.4% for >2 copies [3,28,46]. CN >4 has been reported only among South Americans [3,28,46].

In this article, we investigated and compared how different HNA-1 genotyping methods perform in patients with novel (unreported) or low-frequency variants and with different CNs of *FCGR3B*. This study was conducted to compare the capabilities and limitations of existing technologies with those of a long-read nanopore sequencing method for HNA-1 genotyping. The capabilities and limitations of five different HNA-1 genotyping tests were assessed, including an in-house TaqMan PCR, a commercial PCR-SSP test, PCR-sequence-based typing (PCR-SBT), multiplex ligation-dependent probe amplification (MLPA) and a new assay developed for long-read nanopore sequencing.

## 2. Results

### 2.1. In-House TaqMan PCR Genotyping Data

Five different methods for the detection of HNA-1 variants were compared to test for advances and limitations; a comparison is shown in Table 2. The in-house TaqMan PCR assays were designed to investigate five SNPs (c.114, c.194, c.233, c.244 and c.316) [47]. All the assays also targeted *FCGR3A.* For samples with *FCGR3B* deficiency, the genotyping results are displayed as the homozygosity for the *FCGR3A* nucleotides. For hemizygotes, one nucleotide from *FCGR3B* was measured, while *FCGR3A* appeared as the secondary allele. Genotyping of the five SNPs was successful for all samples with two copies, where the specificity for *FCGR3A* did not influence the result.

**Figure 1:**
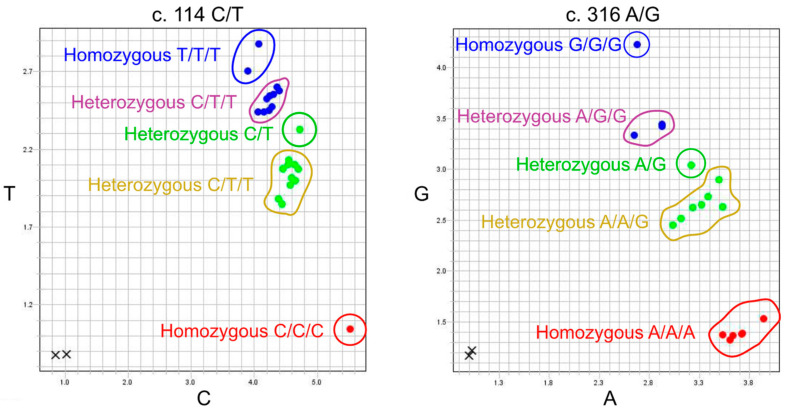
TaqMan cluster plot analysis for c.114C/T and c.316A/G, both showing indication of five clusters. Note: Highlighted blue and red circles are the homozygous clusters, and green circles are the heterozygous cluster representing a sample with two copies. In the spaces between are two intermediate clusters highlighted with purple and yellow representing clusters with heterozygosity but containing three nucleotides.

In samples with three or more copies, the two-dimensional representation of the TaqMan signal highlighted limitations of the assay and revealed the formation of groups distant from the groups with two copies for c.114 and c.316. The amplification plots are shown in Figure 1. The plots show formation of two intermediate clusters (marked with purple and yellow) confirmed to belong to individuals being heterozygotes with three copies (AAB or ABB) in the spaces between the two homozygous clusters (AA/AAA or BB/BBB) (marked with blue and red) and the heterozygote (AB) cluster with two copies (marked with green). There were no observed differences in the homozygous clusters regarding having two or three identical nucleotides. However, there is a risk of incorrect HNA-1 allele assignment resulting in the reporting of only two of the three copies. The in-house TaqMan PCR assay identified rare HNA-1 variants but only variants produced by the five already established SNPs, and it was not possible to determine whether these variants were a result of CNV or specificity for *FCGR3A* without further investigation.

### 2.2. Commercial PCR-SSP Genotyping Dats

The commercial PCR-SSP targeted two of the known SNPs, namely, c.194A (FCGR3B*01), c.233C (FCGR3B*02) and c.233A (FCGR3B*03), as shown in Table 2. In samples with hemizygosity, the results incorrectly show homozygosity, which leads to problems in family studies, as shown in Table 3, where a FCGR3B*Null allele is segregated from a mother to her two children. The commercial PCR-SSP genotyped the mother and child 1 as homozygous for FCGR3B*01, while child 2 was genotyped as homozygous for FCGR3B*02, complicating the line of succession. Copy numbers were determined with real-time quantitative PCR (qPCR), and they were in accordance with copy numbers obtained with MLPA. The HNA-1 genotyping performed with in-house TaqMan PCR and MLPA were also in accordance.

In samples with three copies, three different alleles (e.g., FCGR3B*02, *03, *04) were identified, but samples with multiple copies of the same allele could not be quantified (e.g., FCGR3B*01, *02, *02). The kit was also unable to identify novel or rare variants.

### 2.3. Multiplex Ligation-Dependent Probe Amplification Data

MLPA was designed by the producent to detect CNs, and the assay contains six probes for CN detection in *FCGR3B* and four SNPs for HNA-1 genotyping (c.114, c.194, c.233 and c.316); see Table 2. Dosage quotient analysis was utilized to quantify the exact ratio of each nucleotide for the targeted SNPs. This approach was especially beneficial for samples with three or more copies, but because the MLPA design only genotyped the alleles of four SNPs, the identification of rare or novel variants was difficult/not possible. The CNs obtained with MLPA for both *FCGR3A* and *FCGR3B* were in full agreement with the ones obtained with qPCR, as also previously seen in [12].

### 2.4. PCR-SBT Data

The tested PCR-SBT assay was a previously published assay [48]. Neither this assay nor the assay used by National Health Service Blood and Transplant (NHSBT) in Bristol, United Kingdom was *FCGR3B* specific due to co-amplification of *FCGR3A*, as shown in Table S1. Three replicate samples with three copies of *FCGR3B* showed significant inconsistencies, when tested with the PCR-SBT assay from [48], as shown in Table 4 and Table S2. The three samples were confirmed to have three copies with qPCR. Two of them which where genotyped as FCGR3B*01/*01/*02 and FCGR3B*01/*02/*02 by MLPA were both wrongly genotyped to be FCGR3B*02/*Null by PCR-SBT. The third sample genotyped as FCGR3B*02/*03/*04 by MLPA and long-read sequencing, was genotyped as FCGR3B*04/*Null with PCR-SBT. In the case of c.114 and c.244, they can both correctly be determined as heterozygous, but it is not possible to determine the correct dosage quotient for the nucleotides. For c.194 and c.316, the problem is more severe because the test fails to detect one of the nucleotides, and therefore, presents the SNP as homozygous. No problems were observed for c.233 except the missing of the correct dosage quotient. In principle, this method can be used to find rare variants or novel alleles in the targeted region of *FCGR3B* because it identifies every SNP located in exon 3 in the sequence (526 bp), but the problems with obtaining specificity in this area complicate the use of this method, as it is not possible to definitively assign only to the target gene.

### 2.5. Long-Read Nanopore Sequencing Data

To overcome the limitations of TaqMan PCR, PCR-SSP and PCR-SBT, a long-read product targeting all five exons and flanking introns of *FCGR3B* was designed and sequenced with third-generation sequencing technology (Oxford Nanopore Technologies, Oxford, UK). The primers used were designed to cover the coding part of the *FCGR3B* gene and specifically target *FCGR3B*. Initially, 16 Danish samples with CNs ranging from one to four with rare variants or suspected novel variants were sequenced. To validate the long-read sequencing method, we used an additional validation cohort obtained from the NHSBT. The validation cohort consisted of ten samples previously geno- and phenotyped at the NHSBT containing samples with CNs ranging from zero to three. The ten British samples were analyzed in a blinded experiment at the Department of Clinical Immunology, Aalborg University Hospital. The genotyping results with long-read sequencing of 23 out of 26 samples are shown in Table 5, and in more detail in Tables S3 and S4. The specificity for *FCGR3B* was assessed at five SNPs located in exons 4 and 5 that differed between the *FCGR3A* and *FCGR3B* sequences (rs71632959, rs71632958, rs200215055, rs758550229 and rs374752953 in *FCGR3B*), including the most important difference between the two genes; a C to T change at c.733 (old nomenclature) in *FCGR3B* that creates a stop codon. The assay correctly determined all known *FCGR3B* alleles including the FCGR3B*01, *02, *03, *04 and *05 and was able to genotype samples with zero to four copies; see Table 5. The allelic distribution in samples with three or four copies indicated the possibility of quantification of CNs based on the nucleotide ratio with a ~1:3 (33%:66%) ratio in samples with three copies and a ~1:4 (25%:75%) ratio in samples with four copies; see Table 5. The quantified CNs were consistent with CNs determined with MLPA and qPCR in all tested samples. One hemizygote sample (BRI_008) was tested, and it appeared to be homozygote despite showing lower percentages. In four samples (AAL_004, AAL_007, AAL_009 and AAL_010), heterozygosity for a synonymous mutation (rs368410676) in exon 3 at c.297G/T was observed; see Table S3. This variant was linked to the FCGR3B*05 allele. We found full concordance with previous results from the NHSBT, including a novel allele. In three out of the 26 samples (AAL_003, AAL_013 and BRI_007), two novel alleles were identified, which will be presented in the next section.

### 2.6. Novel Alleles

**Figure 2:**
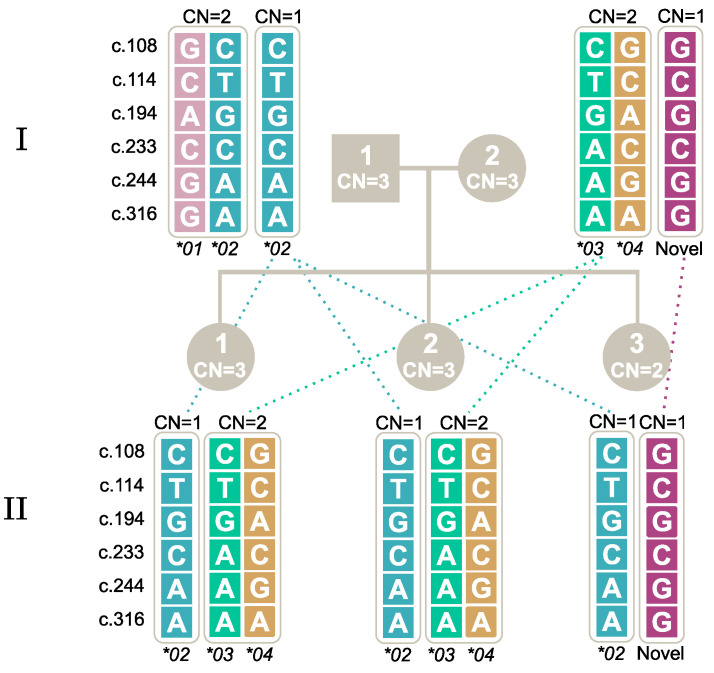
Segregation of a novel *FCGR3B* allele found in AAL_003 (I-2) to AAL_013 (II-3). Note: The first generation is marked with **I** and the second with **II**. Male individuals are marked by square symbols, and females by circles. The individuals’ copy numbers (CN) are shown inside the symbols. The five SNPs and their genotypes are shown for each allele. Alleles inherited together are positioned inside the same bracket.

Two novel alleles not previously described were identified in this study; see Table 6. The first allele was found in a Danish individual (AAL_003) and had the genotype combination of c.108G, c.114C, c.194G, c.233C, c.244A and c.316A, which is identical to the known nucleotide composition of *FCGR3A* except at c.244, as shown in Table 7. Investigations with all described techniques, including long-read sequencing, confirmed the specificity of *FCGR3B*. Additionally, the family study confirmed the inheritance of the novel allele from the mother (AAL_003) to the daughter (AAL_013), as seen in Figure 2.

**Figure 3:**
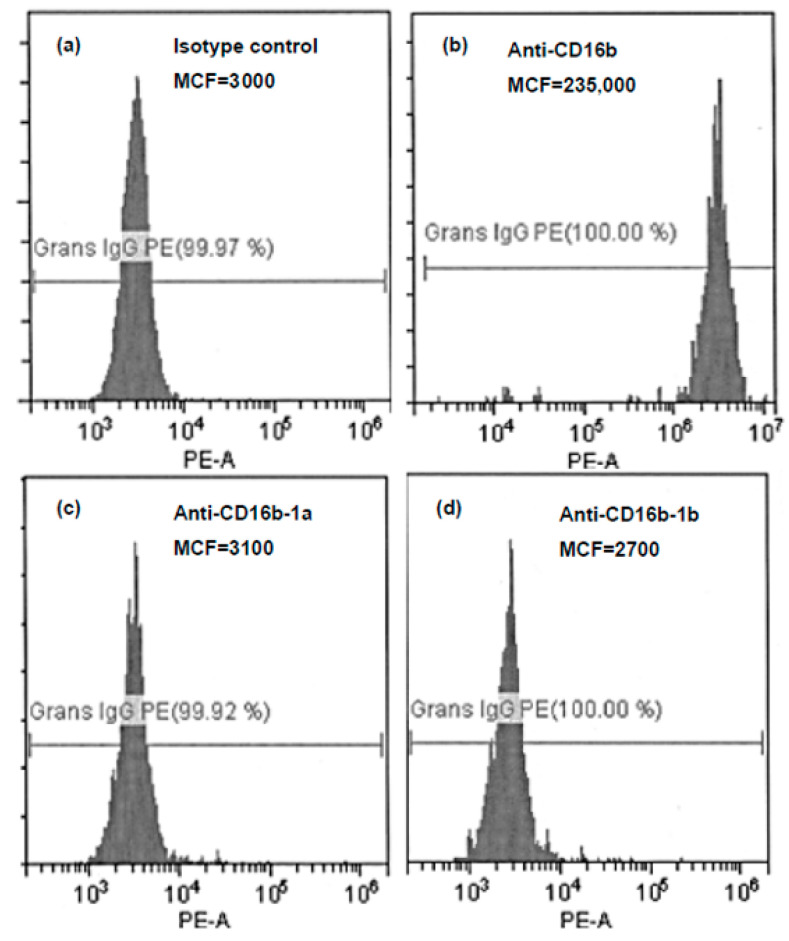
Neutrophil phenotyping with human antisera for the blood donor with a novel HNA-1 (*FCGR3B*) allele (BRI_007). Note: Previously presented by Browne, T., 2022 [49]. (**a**) Isotype control and known negative antisera. (**b**) GI 10 in-house CD16b-specific antisera. (**c**) GI 21 in-house HNA-1a-specific antisera. (**d**) GI 2 in-house HNA-1b-specific antisera. All murine monoclonal antibodies and human antisera specific for CD16b were positive. All CD16b-1a and CD16b-1b human antisera and CD16b-1a monoclonal antibody were negative.

The second novel allele was identified in a hemizygote sample from an English blood donor (NHSBT, BRI_007), which was identical to the *FCGR3A* nucleotide composition at all six SNPs: c.108G, c.114C, c.194G, c.233C, c.244G and c.316A, but had an additional SNP at c.197C; see Table 6 and Table 7. Phenotyping with both human polyclonal and murine monoclonal antibodies at NHSBT showed that the donor neutrophils expressed *FCGR3B* but did not show HNA-1a or HNA-1b specificity, as seen in Figure 3 [49]. The results were reproduced in the testing of a second, subsequent donation sample and confirmed by an external reference as described in [49]. The genotype was then retested via long-read sequencing in a blinded experiment as one of ten samples at the Department of Clinical Immunology, Aalborg University Hospital. Using real-time qPCR, the CN of *FCGR3B* in this sample was determined to be one, and the CN of *FCGR3A* was determined to be two.

### 2.7. Segregation of Alleles through Family Studies

**Figure 4:**
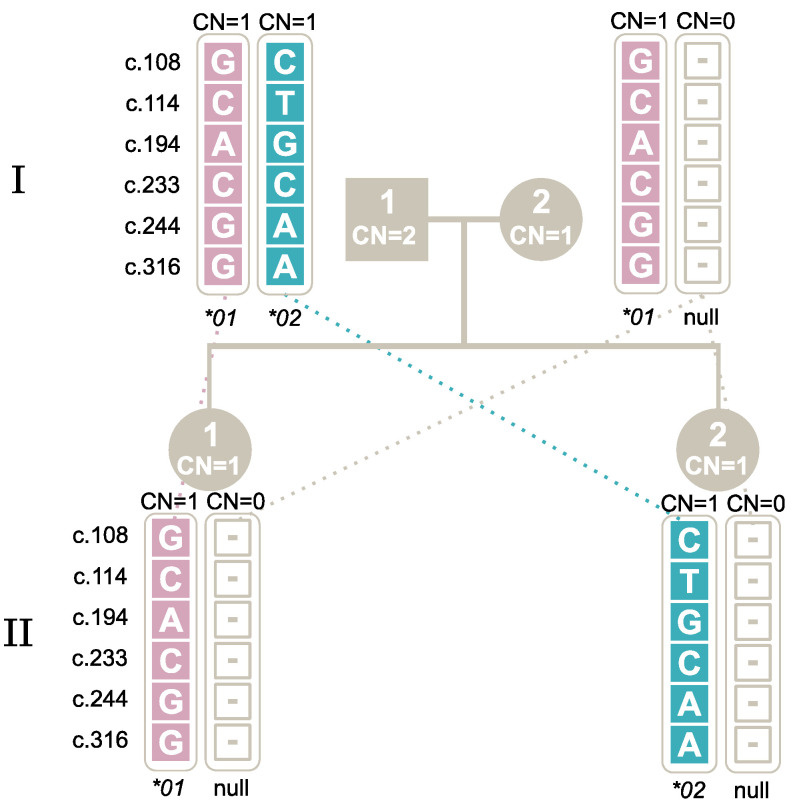
Study I: Segregation of the FCGR3B*Null allele within two generations. Note: The first generation is marked with **I** and the second with **II**. Male individuals are marked by square symbols, and females by circles. The individuals’ copy numbers (CN) are shown inside the symbols. The five SNPs and their genotypes are shown for each allele.

To understand the segregation of rare and numerous *FCGR3B* alleles among Danes, four family studies were carried out, and the different described techniques were applied for HNA-1 genotyping. In Study I, the segregation of a null allele was investigated, as shown in Figure 4, where the null allele was inherited from the mother to both offspring. This family is the one also presented in Table 3. The two offspring inherited different alleles from the father, resulting in the genotypes FCGR3B*01/*Null and FCGR3B*02/*Null. We found that when using a simple technique such as the PCR-SSP commercial kit, which tests only for the presence of FCGR3B*01, FCGR3B*02 and FCGR3B*03, the line of inheritance did not match. The mother who was FCGR3B*01/FCGR3B*Null was wrongfully typed as an FCGR3B*01 homozygote, and therefore, was not able to be the mother of one of her children who was typed as an FCGR3B*02 homozygote (FCGR3B*02/FCGR3B*Null).

**Figure 5:**
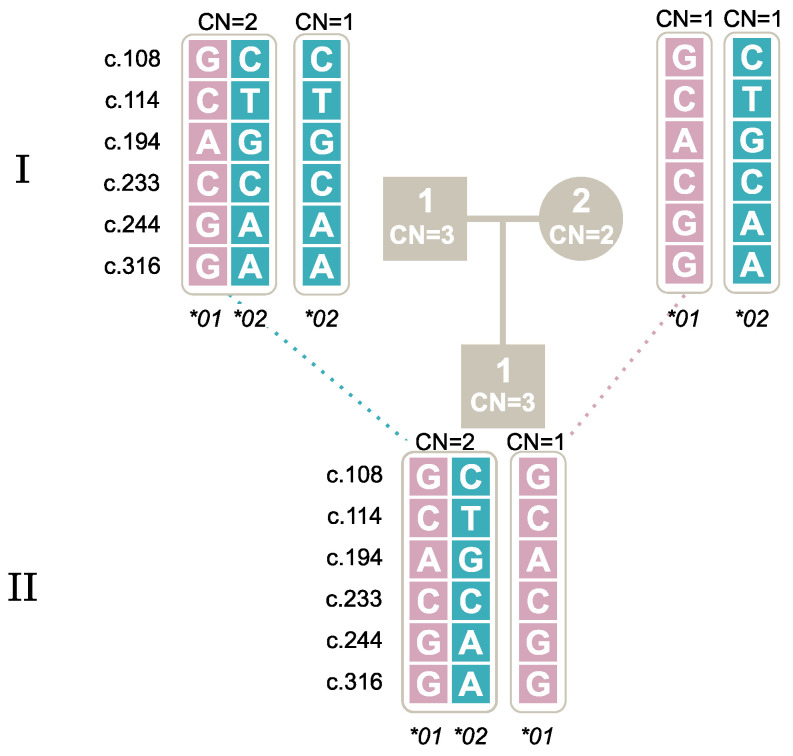
Study II: Segregation of the FCGR3B*01 and FCGR3B*02 together through two generations. Note: The first generation is marked with **I** and the second with **II**. Male individuals are marked by square symbols, and females by circles. The individuals’ copy numbers (CN) are shown inside the symbols. The five SNPs and their genotypes are shown for each allele. Alleles inherited together are positioned inside the same bracket.

Study II investigated which alleles were inherited together in individuals with multiple copies of FCGR3B*01 and FCGR3B*02, shown in Figure 5, where the father had three *FCGR3B* copies, one copy of FCGR3B*01 and two copies of FCGR3B*02. Genotyping of the offspring revealed that one copy of FCGR3B*01 was inherited together with one copy of FCGR3B*02, suggesting that they are located on the same chromosome.

**Figure 6:**
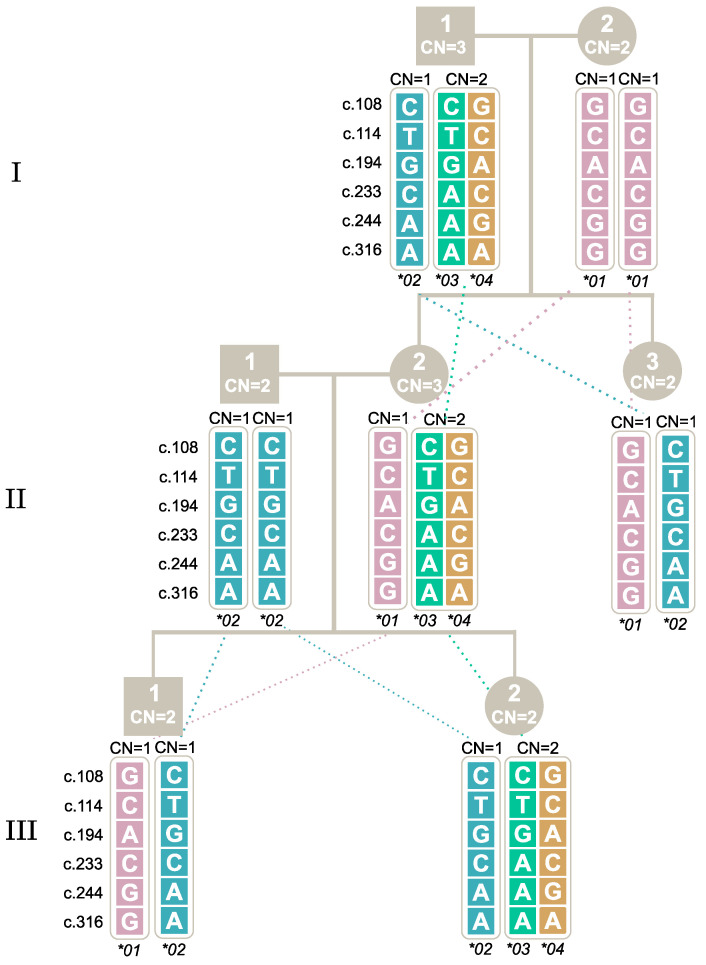
Study III: Segregation of the FCGR3B*03 and FCGR3B*04 together through three generations. Note: The first generation is marked with **I** and the second with **II**. Male individuals are marked by square symbols, and females by circles. The individuals’ copy numbers (CN) are shown inside the symbols. The five SNPs and their genotypes are shown for each allele. Alleles inherited together are positioned inside the same bracket.

Similarly, in Study III, three generations were investigated to determine how the FCGR3B*03 and FCGR3B*04 alleles are linked and inherited together, as shown Figure 6. Previous studies [13] have shown that the FCGR3B*03 and FCGR3B*04 alleles are segregated together, and we also confirmed this to be the case among Danes.

**Figure 7:**
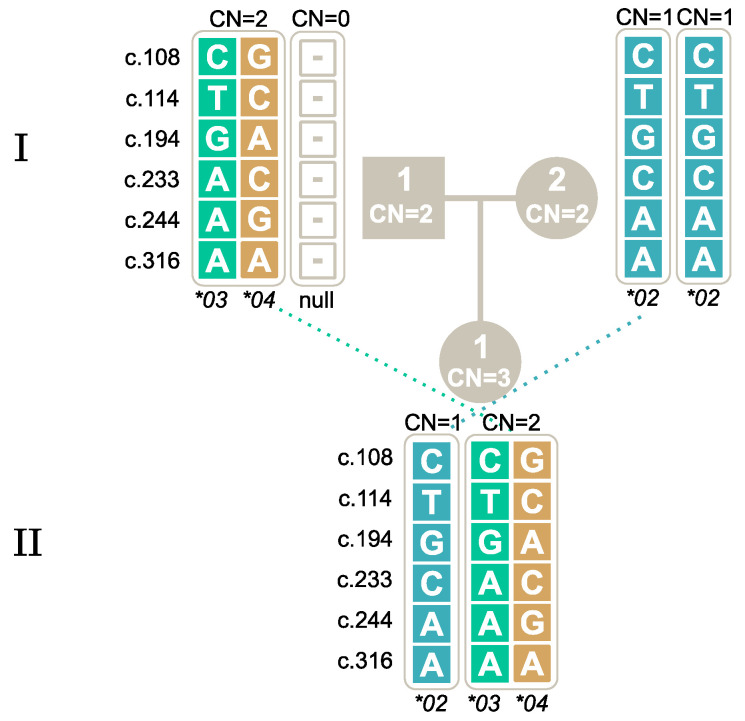
Study IV: Segregation of the FCGR3B*03 and FCGR3B*04 together. Note: The first generation is marked with **I** and the second with **II**. Male individuals are marked by square symbols, and females by circles. The individuals’ copy numbers (CN) are shown inside the symbols. The five SNPs and their genotypes are shown for each allele. Alleles inherited together are positioned inside the same bracket.

Study IV also confirmed the link between FCGR3B*03 and FCGR3B*04 and illustrated how two individuals with two copies can have of an offspring with three copies due to two alleles on the same chromosome and no alleles on the other chromosome; see Figure 7.

## 3. Discussion

Accurate determination of *FCGR3B* alleles is important for the investigation of TRALI, suspicion of neonatal alloimmune neutropenia and confirmation of autoimmune neutropenia during infancy. In this study, five different methods for genotyping *FCGR3B* allelic genotypes were compared. Genotyping of the *FCGR3B* gene is complicated by altered CNs and high homology to the *FCGR3A* gene. The possibility of lower or greater numbers of alleles results in a dosage-dependent distribution, which can lead to ambiguous results when genotyping HNA-1 using TaqMan PCR, PCR-SSP or PCR-SBT assays. TaqMan PCR, our own published in-house assay [47], and a commercial PCR-SSP kit (BAG Diagnostics, Lich, DE, Germany) were not specific for *FCGR3B* because they coamplified the homologous *FCGR3A* gene. As a result, all null alleles are assigned when only the known *FCGR3A* sequence is observed. The TaqMan PCR and PCR-SSP assays proved to be robust in identifying *FCGR3B* variants in samples with one or two copies. TaqMan PCR can indicate the presence of three copies, but not provide full genotypes, while it is unsuitable for samples with more than three copies. When testing samples with higher CNs, c.114 and c.316 showed five clusters instead of three clusters, suggesting that the TaqMan PCR assay has the potential to identify CN alterations. However, this grouping is concentration dependent, and due to obscurities, the higher CNs are reflected in the analysis of the other SNPs, with a risk of interpretation of an incorrect genotype, indicating that this method cannot be used in isolation. As the assays are not specific for *FCGR3B* but also target *FCGR3A,* one would expect that different CNs of *FCGR3A* could influence the results in the same way as different CNs do for *FCGR3B*. CNV in *FCGR3A* is, however, not as frequent as that in *FCGR3B,* and 94% of Europeans have been found to have two copies of *FCGR3A* [15].

We also tested a commercial PCR-SSP kit that tested for only two SNPs, c.194 and c.233, in three reactions (c.194A, c.233C, c.233A); see example in Table 3. This kit can both be used for real-time PCR and gel electrophoresis and provides a positive or negative result. The assay is suitable for high-throughput testing and works for the most frequent variants, such as FCGR3B*01, FCGR3B*02 and FCGR3B*03, but is not able to identify rare variants or novel alleles or provide a dosage-dependent result that would indicate CN alterations. However, it can identify three unique variants (e.g., FCGR3B*01, FCGR3B*02 and FCGR3B*03) present in the same sample.

The MLPA is designed to test for variants and CN alterations in the whole *FCGR2/3* locus, but for *FCGR3B*, it especially investigates six CN probes and four SNPs (c.114, c.194, c.233 and c.316). The amplification products were analyzed with GeneScan, so it was possible to obtain a dosage-dependent quantification of the amplicons. Therefore, this method makes it possible to estimate the number of each nucleotide per SNP, which is especially beneficial for the correct determination of samples with higher CNs. As with the two PCR-SSP assays, this method cannot find novel variants outside of the investigated SNPs. A clinical setting MLPA will be sufficient for most cases. Only in rare cases of novel alleles based on novel SNPs not included in the assay, investigation with other techniques can be relevant.

Investigation outside of the six SNPs defined in the ISBT nomenclature requires sequencing more of the gene. PCR-SBT could be a suitable solution to this, and we tested a published assay targeting exon 3 with a product of 526 bp [48]. This assay was not only specific for *FCGR3B* but also targeted *FCGR3A*, which complicated the genotyping of especially higher CNs. Similar to PCR-SSP, PCR-SBT is a two-dimensional analysis and has complications in regard to dosage dependence. Because of the high homology between *FCGR3B* and *FCGR3A*, we were not able to design a PCR-SBT assay that is gene specific. The in-house PCR-SBT used by NHSBT is also not *FCGR3B* specific.

Long-read sequencing was performed with Nanopore technology, and the results were specific for *FCGR3B*. This method provides dosage-dependent results, which makes it possible to interpret CNs and multiple variants from the results. The method also makes it possible to identify novel SNPs (like c.197A/C in BRI_007) outside of the defined six SNPs in the nomenclature. The only limitation of this method is the ability to detect hemizygosity, as shown in Table 5 and Table 6.

In summary, for most samples with one or two copies, the use of TaqMan PCR and PCR-SSP assays is sufficient, and this is by far the cheapest solution with the simplest throughout. However, we recommend always testing for copy numbers of *FCGR3B* with qPCR, or alternatively MLPA, to identify samples with three or more copies. For special cases that demand further investigation of novel SNPs in *FCGR3B*, different from the six already defined in the ISBT nomenclature, the only functioning methods were long-read nanopore sequencing. Like MLPA, this method is more costly than the PCR-based methods, but also provides more knowledge. In general, in a clinical setting, the PCR-based methods are sufficient for most samples, but in special cases, it can be necessary to carry out further testing or have the samples tested at a laboratory with a more advanced setup.

The family studies identified an allele that did not match any of the alleles described in the ISBT nomenclature. The allele included c.108G, c.114C, c.194G, c.233C, c.244A and c.316A, which means that it was identical to *FCGR3A* except at c.244. The five SNPs in exons 4 and 5, which differ between the *FCGR3A* and *FCGR3B* sequences (rs71632959, rs71632958, rs200215055, rs758550229 and rs374752953 in *FCGR3B*), including the stop codon at c.733 (old nomenclature), confirmed that this was indeed an *FCGR3B* allele; the high resemblance to *FCGR3A* made it difficult to interpret with the PCR-SSP techniques that also had specificity for *FCGR3A,* and since c.244 was not included in the MLPA assay, this method also failed to correctly genotype this sample. The complexity of the testing did not decrease because the first tested sample (AAL_003) had three copies of *FCGR3B*, the other two being FCGR3B*03 and FCGR3B*04. We therefore tested segregation to the next generation and found that two offspring inherited FCGR3B*03 and FCGR3B*04, while the last (AAL_013) inherited the same new allele. The similarity to the allelic composition of *FCGR3A* provides difficulties in genotyping this allele and could explain why it has not been previously described.

A similar case was found in a British sample (BRI_007) originally genotyped at NHSBT in the United Kingdom, and the findings were replicated at two other laboratories, including our laboratory. This sample was identical to that of *FCGR3A* at all six SNPs, c.108G, c.114C, c.194G, c.233C, c.244G and c.316A, but had a novel mutation at c.197 resulting in the substitution of a Leu with an Arg at p.66. As for the Danish sample, it was confirmed with the five SNPs in exons 4 and 5 that it was indeed an *FCGR3B* allele. Phenotyping studies revealed that CD16b was expressed on the neutrophils of this donor, but cells were negative for HNA-1a and HNA-1b. These findings are consistent with a recently reported study in which four key aa’s that differed between the two variants (1a and 1b) were altered in transfected cells [50]. This study showed that the epitope formation of HNA-1a only depended on p.Asn65, while the formation of HNA-1b depended on p.Ser36 and/or p.Asn82. Based on these findings, the British allele (BRI_007) containing p.Ser65, p.Arg36 and p.Asp82 has neither HNA-1a nor HNA-1b epitopes, while the novel Danish allele (AAL_003 and AAL_013) containing p.Ser65, p.Arg36 and p.Asn82 could have an HNA-1b epitope. Phenotyping was not performed for the novel Danish allele, as it was found in combination with either FCGR3B*02 or FCGR3B*03, both of which have p.Asn82.

The two novel variants were not identified with current, routine testing. The potential consequence of this is that they and others like them could have been wrongly interpreted in the past and that these types of variants exist in other patients and donors but are overlooked with the current reliance on inferred expression based on the current genotyping method. For definite allele assignment, we therefore recommend long-read nanopore sequencing.

## 4. Methods

### 4.1. Study Cohort

Thirty-eight samples from patients and family members who had previously been genotyped for HNA-1 for primary autoimmune neutropenia investigation at the Department of Clinical Immunology, Aalborg University Hospital, Denmark, were selected for this study. The Department of Clinical Immunology is the national center for diagnostic neutrophil testing in Denmark and was the center for sample collection. DNA was extracted from EDTA-stabilized whole blood or buccal swabs using the Maxwell RSC Blood DNA Kit or the Maxwell RSC Buccal Swab Kit on the Maxwell RSC instrument according to the manufacturer’s protocol (Promega, Madison, WI, USA). Written and oral informed consent from the participants was obtained according to the Danish Health Care Act and in accordance with the Declaration of Helsinki. The study was approved by the North Denmark Region Committee on Health Research Ethics (approval number: N-20170026).

### 4.2. Validation Cohort

Ten DNA samples from consented English blood donors who were originally geno- and phenotyped at NHSBT in Bristol, United Kingdom, were collected and tested at the laboratory at the Department of Clinical Immunology, Aalborg University Hospital, Denmark, as a blinded experiment for method verification. DNA was extracted at NHSBT either automated using the MagnaPure Nucleic Acid Isolation Kit I on a MagnaPure Compact (Roche, Basel, CH) or manual with the Puregene Blood Kit (Qiagen, Hilden, DE, Germany) according to each manufacturer’s protocol. Granulocytes were isolated, fixed and phenotyped for HNA-1 (CD16b) expression following existing in-house protocols (monoclonal 3G8, in-house polyclonal GI10, GI11) at NHSBT. Additional antigen-specific phenotyping was performed on selected samples using human polyclonal antibody reference serum for HNA-1a (NIBSC reference and in-house GI21, GI23) and HNA-1b (in-house GI2 and GI17). Genotyping for HNA-1 was performed by PCR-SBT following a validated in-house protocol optimized from the method described by Culliford et al. [51], which provides a 594 bp amplicon covering exon 3; see primers in Table S1.

### 4.3. In-House TaqMan PCR and Commercial PCR-SSP Testing

Genotyping of *FCGR3B* was performed with an in-house TaqMan PCR genotyping assay targeting five SNPs (rs527909462, rs448740, rs5030738, rs147574249 and rs2290834) previously published in [47] and shown in Table S5. The specificity of the primers was tested with Ensembl [52]. DNA amplification was carried out in a 20 µL volume containing 20 ng of DNA, 0.9 µM primers and 0.2 µM probes (final concentrations) amplified in 96-well plates. PCRs were performed with the following protocol on a Proflex (Applied Biosystems, Waltham, MA, USA): 95 °C for 10 min, followed by 40 cycles of 95 °C for 15 s and 60 °C for 1 min. Subsequently, endpoint fluorescence was determined using QuantStudio 12K Flex software v1.4 on a QuantStudio 12K Flex platform (Applied Biosystems, Waltham, MA, USA). The in-house *FCGR3B* analysis is routinely performed on patients in Denmark and is ISO accredited (DANAK, DK) and validated continuously in the International Granulocyte Immunology Workshops (IGIW) and INSTAND eV. PCR-SSP genotyping was performed with the commercial ERY Q^®^ HNA Kit from BAG Diagnostics according to the manufacturer’s protocol (BAG Diagnostics, Lich, DE).

### 4.4. Real-Time qPCR CN Investigation

CNs of *FCGR3A* and *FCGR3B* were characterized using qPCR TaqMan Copy Number Assays, *FCGR3A* (Hs00139300_cn) and *FCGR3B* (Hs04211858_cn), and the TaqMan Copy Number Reference Assay, RNase P, according to the manufacturers’ protocol (Applied Biosystems, Waltham, MA, USA) [41].

### 4.5. Multiplex Ligation-Dependent Probe Amplification Testing

MLPA was performed using the SALSA MLPA probe mixes P110-C1 FCGR mix 1 and P111-C1 FCGR mix 2, according to the manufacturer’s protocol (MRC-Holland, Amsterdam, The Netherlands) as previously described [12].

### 4.6. PCR-SBT Analysis

Selected samples were also subjected to PCR-SBT which was performed with primers previously published by J. He et al. [48] targeting exon 3 and providing a 526 bp product; see Table S1. Sequencing was performed using a BigDyeDirect Cycle Sequencing Kit according to the manufacturer’s protocol (Thermo Fisher Scientific, Waltham, MA, USA) on a 3500 Genetic Analyzer (Applied Biosystems, Waltham, MA, USA). For more details of the PCR-SBT typing; see Table S2.

### 4.7. Long-Read Nanopore Sequencing

Primers for *FCGR3B*-specific amplification, shown in Table S6, were designed with CLC Main Workbench 21 (Qiagen, Hilden, DE), and Ensembl [52] was used to confirm that the primers did not target *FCGR3A*. The amplicon (8262 bp) included all five exons and flanking intron in *FCGR3B*. Each 20 µL PCR reaction consisted of 200 ng of template DNA, 10 µL of Long-Range PCR: GoTaq^®^ Long PCR Master Mix (Promega, Madison, WI, USA) and 1 µL of primer mix (final conc. 0.5 mM of each). PCR was performed on a Proflex™ (Applied Biosystems, USA) with the following conditions: 95 °C for 2 min, 35 cycles of 94 °C for 30 s, 53 °C for 30 s and 65 °C for 9 min, and one cycle of 72 °C for 10 min. PCR products were visualized via gel electrophoresis and quantified with a Qubit FlexFluorometer (Thermo Fisher Scientific, Waltham, MA, USA) using a Broad Range Kit (Thermo Fisher Scientific, Waltham, MA, USA). Barcoding and sequencing were performed with the Rapid Sequencing gDNA-Barcoding protocol (SQK-RBK110.96) (version RBK_9126_v110_revO_24Mar2021) (Oxford Nanopore, Oxford, UK). Two hundred nanograms of amplification product was diluted to a volume of 7.5 µL, and 2.5 µL of barcoding mix was added according to the manufacturer’s protocol (Oxford Nanopore, Oxford, UK). The samples were cleaned with AMPure XP (Beckman Coulter, Brea, CA, USA) beads before pooling. Sequencing was performed with an R9.4 flow cell on a MinION 1 kb sequencer (Oxford Nanopore, Oxford, UK). Basecalling was performed with guppy alignment software v. 6.1.5, sequencing files were aligned to the human genome reference (Hg38) using minimap2 v. 2.22 and indexing was performed with the SAMtools index. Manual analysis was performed using Integrated Genomics Viewer (IGV) v. 2.16.1.

## Figures and Tables

**Table 1:** Overview of HNA-1 alleles including the six defined SNPs in the ISBT nomenclature in exon 3 of *FCGR3B* and five additional SNPs in exon 4 and 5 that differs between *FCGR3A* and *FCGR3B*.

New Nomenclature	c.108	c.114	c.194	c.233	c.244	c.316					STOP CODON
Old Nomenclature	c.141	c.147	c.227	c.266	c.277	c.349	c.473	c.505	c.559	c.641	c.733
rs no.	rs200688856	rs527909462	rs448740	rs5030738	rs147574249	rs2290834	rs71632959	rs71632958	rs200215055	rs758550229	rs374752953
Pos (Hg38)	161,629,989	161,629,983	161,629,903	161,629,864	161,629,853	161,629,781	161,626,282	161,626,250	161,626,196	161,624,609	161,624,517
Exons in *FCGR3B*	3						4			5	
Amino acid change	Arg36Ser	38Leu	Asn65Ser	Ala78Asp	Asp82Asn	Val106Ile			Val176Phe		
FCGR3B*01	G	C	A	C	G	G	A	C	G	C	T
FCGR3B*02	C	T	G	C	A	A	A	C	G	C	T
FCGR3B*03	C	T	G	A	A	A	A	C	G	C	T
FCGR3B*04 (FCGR3B*01 var c.349G)	G	C	A	C	G	A	A	C	G	C	T
FCGR3B*05 (FCGR3B*02 var c.244G)	C	T	G	C	G	A	A	C	G	C	T
FCGR3B*01 var c.194G	G	C	G	C	G	G	A	C	G	C	T
FCGR3B*02 var c.194G	C	T	A	C	A	A	A	C	G	C	T
*FCGR3A*	G	C	G	C	G	A	G	T	T	T	C

**Table 2:** Comparison of methods for HNA-1 genotyping.

Method	Type	SNPs Included						CN Included	*FCGR3B* Specific	Detect Hemizygosity ^1^	Type >2 Alleles	Detect Rare Variants
		c.108	c.114	c.194	c.233	c.244	c.316					
In-house	TaqMan PCR		x	x	x	x	x	No	No	Yes/no ^2^	No	Yes/no ^3^
ERY Q	PCR-SSP/Gel			x	x			No	Yes	No	No	No
MLPA	Genescan		x	x	x		x	Yes	Yes	Yes	Yes	Yes/no ^3^
Sanger sequencing	PCR-SBT	Short fragment sequencing of exon 3 (526 bp)						No	No	No	No	No
Nanopore sequencing	Target specific amplification	Long-read sequencing (8262 bp)						Yes ^4^	Yes	No ^5^	Yes	Yes

^1^ Null alleles are not amplified, so null/null individuals will not produce amplification products in any of the tests. ^2^ Null alleles allow the detection of *FCGR3A* alleles. ^3^ Limited to variants in the investigated SNPs. ^4^ Copy number can be estimated based on the percentage distribution of alleles. ^5^ Individuals with one copy are seen as homozygotes.

**Table 3:** Comparison of PCR-SSP, qPCR, TaqMan PCR and MLPA test results and conclusion in a family with hemizygosity.

	Commercial PCR-SSP ^1^	qPCR	In-House TaqMan PCR	MLPA
Father	c.194A(+) c.233C(+) c.233A(-)	CN = 2	c.114C/T c.194A/G c.233C/C c.244G/A c.316G/A	CN = 2 c.114C/T c.194A/G c.233C/C c.316G/A
	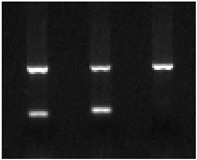			
	FCGR3B*01/*02		FCGR3B*01/*02	FCGR3B*01/*02
Mother	c.194A(+) c.233C(-) c.233A(-)	CN = 1	c.114C/C c.194A/G c.233C/C c.244G/G c.316G/A	CN = 1 c.114C c.194A c.233C c.316G
	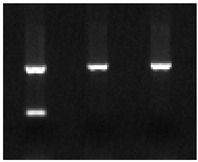			
	FCGR3B*01		FCGR3B*01/*Null ^2^	FCGR3B*01/*Null
Child 1	c.194A(+) c.233C(-) c.233A(-)	CN = 1	c.114C/C c.194A/G c.233C/C c.244G/G c.316G/A	CN = 1 c.114C c.194A c.233C c.316G
	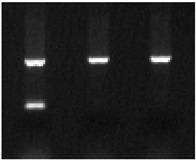			
	FCGR3B*01		FCGR3B*01/*Null ^2^	FCGR3B*01/*Null
Child 2	c.194A(-) c.233C(+) c.233A(-)	CN = 1	c.114T/C c.194G/G c.233C/C c.244A/G c.316A/A	CN = 1 c.114T c.194G c.233C c.316A
	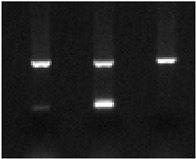			
	FCGR3B*02		FCGR3B*02/*Null ^2^	FCGR3B*02/*Null

^1^ Top band is control band, positive outcome is determined based on the second band. ^2^ Interpretated as the *FCGR3A* (CGCGA) allelic genotype.

**Table 4:** Overview of PCR-SBT results for samples with three copies compared to typing results confirmed with MLPA and long-read sequencing. Copy numbers of three samples and HNA-1 variants have been confirmed with qPCR and MLPA.

	*Test*	*Genotype*	c.114	c.194	c.233	c.244	c.316
Sample 1	*MLPA*	FCGR3B*01/*01/*02	CCT	AAG	CCC	GGA	GGA
	*PCR-SBT*	FCGR3B*02/*Null	CT	GG	CC	GA	AA
Sample 2	*MLPA*	FCGR3B*01/*02/*02	CTT	AGG	CCC	GAA	GAA
	*PCR-SBT*	FCGR3B*02/*Null	CT	GG	CC	GA	AA
Sample 3	*MLPA/Nanopore*	FCGR3B*02/*03/*04	TTC	GGA	CAC	AAG	AAA
	*PCR-SBT*	FCGR3B*04/*Null	CT	GG	CA	AG	AA

**Table 5:** Long-read nanopore sequencing results of 23 samples (14 Danish and 9 British).

Sample ID	CN *FCGR3B* ^1^	c.108	c.114	c.194	c.233	c.244	c.316	*FCGR3B* Alleles
AAL_016	0	A: 0% C: 0% G: 0% T: 0%	A: 0% C: 0% G: 0% T: 0%	A: 0% C: 0% G: 0% T: 0%	A: 0% C: 1% G: 0% T: 0%	A: 0% C: 0% G: 0% T: 0%	A: 0% C: 0% G: 0% T: 0%	*Null/*Null
BRI_006	0	A: 0% C: 2% G: 0% T: 0%	A: 2% C: 0% G: 2% T: 0%	A: 1% C: 3% G: 0% T: 0%	A: 2% C: 0% G: 2% T: 0%	A: 2% C: 0% G: 1% T: 0%	A: 4% C: 0% G: 0% T: 0%	*Null/*Null
BRI_008	1	G: 85%	C: 71%	A: 86%	C: 87%	G: 82%	G: 83%	*01/*Null
AAL_001	2	G: 43% C: 51%	T: 55% C: 40%	G: 47% A: 49%	C: 94%	G: 43% A: 53%	G: 44% A: 51%	*01/*02
AAL_011	2	G: 42% C: 52%	T: 58% C: 37%	G: 48% A: 48%	C: 95%	G: 41% A: 54%	G: 43% A: 53%	*01/*02
BRI_001	2	G: 42% C: 51%	T: 57% C: 37%	G: 48% A: 47%	C: 94%	G: 42% A: 53%	G: 43% A: 52%	*01/*02
BRI_004	2	G: 41% C: 50%	T: 56% C: 36%	G: 47% A: 47%	C: 92%	G: 41% A: 52%	G: 42% A: 51%	*01/*02
BRI_005	2	G: 41% C: 50%	T: 56% C: 36%	G: 47% A: 47%	C: 92%	G: 41% A: 52%	G: 42% A: 51%	*01/*02
BRI_003	2	G: 43% C: 50%	T: 56% C: 37%	G: 46% A: 49%	C: 47% A: 47%	G: 42% A: 52%	G: 44% A: 50%	*01/*03
AAL_008	2	C: 92%	T: 93%	G: 91%	C: 49% A: 48%	A: 93%	A: 96%	*02/*03
AAL_005	2	C: 95%	T: 96%	G: 47% A: 52%	C: 97%	A: 98%	A: 97%	*02/*02 var c.194G
AAL_004	2	G: 43% C: 50%	T: 56% C: 38%	G: 46% A: 49%	C: 93%	G: 88%	G: 45% A: 50%	*01/*05 ^2^
AAL_009	2	C: 95%	T: 96%	G: 95%	C: 97%	G: 46% A: 52%	A: 97%	*02/*05 ^2^
AAL_010	2	C: 95%	T: 95%	G: 95%	C: 97%	G: 45% A: 53%	A: 97%	*02/*05 ^2^
AAL_007	2	G: 41% C: 51%	T: 57% C: 37%	G: 47% A: 47%	C: 93%	G: 84%	A: 93%	*04/*05 ^2^
AAL_014	3	G: 56% C: 36%	T: 44% C: 49%	G: 33% A: 61%	C: 60% A: 33%	G: 55% A: 39%	G: 28% A: 65%	*01/*03/*04
AAL_002	3	G: 30% C: 65%	T: 68% C: 27%	G: 62% A: 35%	C: 63% A: 32%	G: 29% A: 67%	A: 96%	*02/*03/*04
AAL_006	3	G: 28% C: 65%	T: 68% C: 25%	G: 61% A: 34%	C: 63% A: 31%	G: 28% A: 66%	A: 93%	*02/*03/*04
AAL_012	3	G: 29% C: 62%	T: 65% C: 26%	G: 59% A: 34%	C: 63% A: 30%	G: 29% A: 64%	A: 94%	*02/*03/*04
BRI_002	3	G: 29% C: 64%	T: 67% C: 26%	G: 60% A: 35%	C: 64% A: 30%	G: 29% A: 66%	A: 92%	*02/*03/*04
BRI_009	3	G: 30% C: 64%	T: 67% C: 27%	G: 60% A: 36%	C: 64% A: 31%	G: 30% A: 66%	A: 94%	*02/*03/*04
BRI_010	3	G: 34% C: 61%	T: 66% C: 30%	G: 57% A: 40%	C: 73% A: 24%	G: 34% A: 63%	A: 83%	*02/*03/*04
AAL_015	4	G: 44% C: 50%	T: 56% C: 39%	G: 46% A: 50%	C: 71% A: 24%	G: 43% A: 52%	G: 24% A: 72%	*01/*02/*03/*04

^1^ Determined with qPCR. ^2^ Heterozygote for a synonymous mutation (rs368410676) in exon 3 at c.297G/T.

**Table 6:** Long-read sequencing results of three samples with novel alleles.

Sample ID	CN *FCGR3B* ^1^	c.108	c.114	c.194	c.197 ^2^	c.233	c.244	c.316	*FCGR3B* alleles
AAL_003	3	G: 61% C: 33%	T: 39% C: 55%	G: 60% A: 36%	A: 95%	C: 65% A: 30%	G: 30% A: 65%	A: 95%	*03/*04/Novel (GCGCAA ^3^)
AAL_013	2	G: 45% C: 51%	T: 57% C: 40%	G: 95%	A: 95%	C: 97%	A: 97%	A: 97%	*02/Novel (GCGCAA ^3^)
BRI_007	1	G: 83%	C: 70%	G: 83%	C: 79%	C: 85%	G: 80%	A: 85%	*Null/Novel (GCGCGA ^3^ + novel SNP c.197C)

^1^ Determined with qPCR. ^2^ This SNP is not included in the six SNPs defined in the ISBT nomenclature and highlighted with a darker background color. ^3^ Allele described as the nucleotides at the six SNPs positions defined in the ISBT nomenclature (c.197A/C not included).

**Table 7:** Novel alleles compared to defined SNPs in the ISBT nomenclature.

Nucleotide	c.108	c.114	c.194	c.197 ^1^	c.233	c.244	c.316					STOP CODON
Pos on chr.1 (Hg38)	161,629,989	161,629,983	161,629,903	161,629,900	161,629,864	161,629,853	161,629,781	161,626,282	161,626,250	161,626,196	161,624,609	161,624,517
rs no. in *FCGR3B*	rs200688856	rs527909462	rs448740	rs761007181	rs5030738	rs147574249	rs2290834	rs71632959	rs71632958	rs200215055	rs758550229	rs374752953
Exons in *FCGR3B*	3							4			5	
Amino acid change	Arg36Ser	38Leu	Asn65Ser	Leu66Arg	Ala78Asp	Asp82Asn	Val106Ile					
FCGR3B*01	G	C	A	A	C	G	G	A	C	G	C	T
FCGR3B*02	C	T	G	A	C	A	A	A	C	G	C	T
FCGR3B*03	C	T	G	A	A	A	A	A	C	G	C	T
FCGR3B*04	G	C	A	C	G	A	G	A	C	G	C	T
FCGR3B*05	C	T	G	A	C	G	A	A	C	G	C	T
FCGR3B*01 var c. 194G	G	C	G	A	C	G	G	A	C	G	C	T
FCGR3B*02 var c. 194A	C	T	A	A	C	A	A	A	C	G	C	T
Novel 1 (AAL_003, AAL_013)	G	C	G	A	C	A	A	A	C	G	C	T
Novel 2 (BRI_007)	G	C	G	C	C	G	A	A	C	G	C	T
*FCGR3A*	G	C	G	A	C	G	A	G	T	T	T	C

^1^ This SNP is not included in the six SNPs defined in the ISBT nomenclature. Novel SNP and novel alleles are highlighted with a darker background color.

## Data Availability

The original contributions presented in the study are included in the article/supplementary material, further inquiries can be directed to the corresponding author/s.

## Supplementary Materials

The following supporting information can be downloaded at: https://www.mdpi.com/article/10.3390/ijms25169127/s1.

## Abbreviations

AA, amino acid; CN, copy numbers; CNR, copy number region; CNV, copy number variation; FcγRIIIb, Fc gamma receptor IIIb; HNA, human neutrophil antigen; IGV, Integrative Genomics Viewer; ISBT, International Society of Blood Transfusion; MLPA, multiplex ligation-dependent probe amplification, NHSBT; National Health Service Blood and Transplant; PCR, polymerase chain reaction; PCR-SBT, PCR-sequence-based typing; PCR-SSP, PCR-sequence-specific primer; qPCR, real-time quantitative PCR; SNP, single nucleotide polymorphism; TRALI, transfusion-related acute lung injury.
