# Supplementary material for: Pitfalls When Determining HNA-1 Genotypes and Finding Novel Alleles
Source: Int J Mol Sci. 2024 Aug 22;25(16):9127. doi: 10.3390/ijms25169127 (PMC11354314; doi:10.3390/ijms25169127)
Supplement: Supplementary file 1 [file ijms-25-09127-s001.zip › ijms-3120767-supplementary.pdf]

Supplementary

Table S1 Primer design PCR-SBT

Region	Sequence	Gen. Loc.
From He 2014 [50]		
Fwd	GGGCCAAGATGCTCTAAGAC	1:161548796-161548815 (FCGR3A)<br>1:161630153-161630172 (FCGR3B)
Rev	CCAGTGGGACCACACATCATC	1:161548290-161548309 (FCGR3A)<br>1:161629647-161629666 (FCGR3B)
In-house NHSBT from Culliford et al. [47]		
	5’-TGAGGCTCCCTGGGAATGGC	1:161548734-161548753 (FCGR3A)<br>1:161630091-161630110 (FCGR3B)

Table S2 PCR-SBT results of samples with three copies

***FCGR3B*01/*01/*02* genotyped as *FCGR3B*02/null*[1]**						
**Allele**	**Epitope**	**c.114**	**c.194**	**c.233**	**c.244**	**c.316**
*FCGR3B*01*	HNA-1a	C	A	C	G	G
*FCGR3B*01*	HNA-1a	C	A	C	G	G
*FCGR3B*02*	HNA-1b	T	G	C	A	A
FCGR3A	HNA-null	C	G	C	G	A
FCGR3A	HNA-null	C	G	C	G	A
PCR-SBT		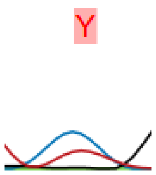	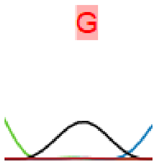	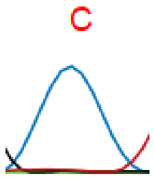	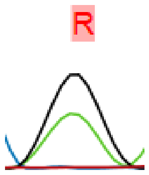	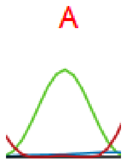
***FCGR3B*01/*02/*02* genotyped as *FCGR3B*02/null*[1]**						

Allele	Epitope	c.114	c.194	c.233	c.244	c.316
*FCGR3B*01*	HNA-1a	C	A	C	G	G
*FCGR3B*02*	HNA-1b	T	G	C	A	A
*FCGR3B*02*	HNA-1b	T	G	C	A	A
FCGR3A	HNA-null	C	G	C	G	A
FCGR3A	HNA-null	C	G	C	G	A
PCR-SBT		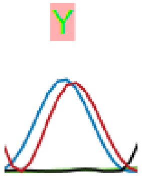	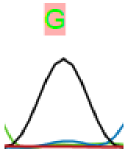	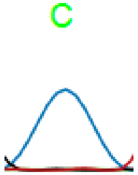	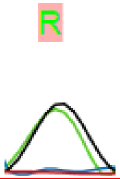	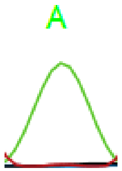
FCGR3B*02/*03/*04 genotyped as FCGR3B*04/null[1]						
**Allele**	**Epitope**	**c.114**	**c.194**	**c.233**	**c.244**	**c.316**
*FCGR3B*02*	HNA-1b	T	G	C	A	A
*FCGR3B*03*	HNA-1c	T	G	A	A	A
*FCGR3B*04*	HNA-1a var	C	A	C	G	A
FCGR3A	HNA-null	C	G	C	G	A
FCGR3A	HNA-null	C	G	C	G	A
PCR-SBT		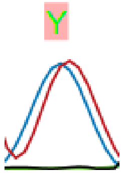	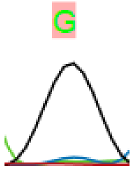	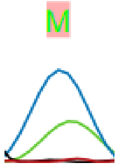	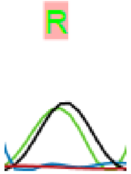	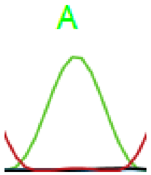

[1]HNA-1 variant and copy numbers determined with MLPA and qPCR

Table S3 Long-read sequencing raw data for 11 SNPs in 16 Danish samples

New	c.108	c.114	c.194	c.233	c.244	c.316					STOP CODON	
Old	c.141	c.147	c.227	c.266	c.277	c.349	c.473	c.505	c.559	c.641	c.733	
Sample ID	rs200688856	rs527909462	rs448740	rs5030738	rs147574249	rs2290834	rs71632959	rs71632958	rs200215055	rs758550229	rs374752953	FCGR3B alleles
	chr1:161.629.989	chr1:161.629.983	chr1:161.629.903	chr1:161.629.864	chr1:161.629.853	chr1:161.629.781	chr1:161.626.282	chr1:161.626.250	chr1:161.626.196	chr1:161.624.609	chr1:161.624.517	
AAL_001	Total count: 4340<br>T: 68 (2%, 58+, 10-)<br>**G: 1861 (43%, 1611+, 250-)**<br>**C: 2221 (51%, 1793+, 428-)**<br>A: 54 (1%, 28+, 26-)<br>N : 136 (3%, 61+, 75-)<br>---------------<br>DEL: 347<br>INS: 70	Total count: 4440<br>**T: 2431 (55%, 2012+, 419-)**<br>G: 67 (2%, 63+, 4-)<br>**C: 1766 (40%, 1444+, 322-)**<br>A: 29 (1%, 20+, 9-)<br>N : 147 (3%, 58+, 89-)<br>---------------<br>DEL: 226<br>INS: 81	Total count: 4537<br>T: 18 (0%, 17+, 1-)<br>**G: 2132 (47%, 1763+, 369-)**<br>C: 10 (0%, 5+, 5-)<br>**A: 2215 (49%, 1803+, 412-)**<br>N : 162 (4%, 72+, 90-)<br>---------------<br>DEL: 112<br>INS: 69	Total count: 4548<br>T: 71 (2%, 66+, 5-)<br>G: 2 (0%, 1+, 1-)<br>**C: 4293 (94%, 3479+, 814-)**<br>A: 12 (0%, 12+, 0-)<br>N : 170 (4%, 78+, 92-)<br>---------------<br>DEL: 92<br>INS: 16	Total count: 4547<br>T: 27 (1%, 22+, 5-)<br>**G: 1938 (43%, 1606+, 332-)**<br>C: 17 (0%, 11+, 6-)<br>**A: 2395 (53%, 1890+, 505-)**<br>N : 170 (4%, 76+, 94-)<br>---------------<br>DEL: 51<br>INS: 83	Total count: 4568<br>T: 14 (0%, 14+, 0-)<br>**G: 2018 (44%, 1627+, 391-)**<br>C: 9 (0%, 6+, 3-)<br>**A: 2351 (51%, 1792+, 559-)**<br>N : 176 (4%, 74+, 102-)<br>---------------<br>DEL: 92<br>INS: 47	Total count: 4323<br>T: 11 (0%, 4+, 7-)<br>G: 81 (2%, 3+, 78-)<br>C: 10 (0%, 2+, 8-)<br>**A: 4201 (97%, 1521+, 2680-)**<br>N : 20 (0%, 12+, 8-)<br>---------------<br>DEL: 56<br>INS: 48	Total count: 4180<br>T: 16 (0%, 6+, 10-)<br>G: 4 (0%, 1+, 3-)<br>**C: 4125 (99%, 1454+, 2671-)**<br>A: 14 (0%, 11+, 3-)<br>N : 21 (1%, 13+, 8-)<br>---------------<br>DEL: 205<br>INS: 90	Total count: 4339<br>T: 6 (0%, 2+, 4-)<br>**G: 4268 (98%, 1502+, 2766-)**<br>C: 10 (0%, 7+, 3-)<br>A: 35 (1%, 12+, 23-)<br>N : 20 (0%, 14+, 6-)<br>---------------<br>DEL: 45<br>INS: 15	Total count: 4521<br>T: 19 (0%, 18+, 1-)<br>G: 3 (0%, 0+, 3-)<br>**C: 4460 (99%, 940+, 3520-)**<br>A: 5 (0%, 0+, 5-)<br>N : 34 (1%, 2+, 32-)<br>---------------<br>DEL: 82<br>INS: 23	Total count: 4524<br>**T: 4468 (99%, 959+, 3509-)**<br>G: 8 (0%, 0+, 8-)<br>C: 11 (0%, 3+, 8-)<br>A: 3 (0%, 2+, 1-)<br>N : 34 (1%, 2+, 32-)<br>---------------<br>DEL: 93<br>INS: 246	*01/*02
	chr1:161.629.989	chr1:161.629.983	chr1:161.629.903	chr1:161.629.864	chr1:161.629.853	chr1:161.629.781	chr1:161.626.282	chr1:161.626.250	chr1:161.626.196	chr1:161.624.609	chr1:161.624.517	
AAL_002	Total count: 5014<br>T: 82 (2%, 69+, 13-)<br>**G: 1506 (30%, 1283+, 223-)**<br>**C: 3253 (65%, 2526+, 727-)**<br>A: 55 (1%, 41+, 14-)<br>N : 118 (2%, 61+, 57-)<br>---------------<br>DEL: 299<br>INS: 91	Total count: 5054<br>**T: 3441 (68%, 2735+, 706-)**<br>G: 73 (1%, 64+, 9-)<br>**C: 1385 (27%, 1098+, 287-)**<br>A: 34 (1%, 15+, 19-)<br>N : 121 (2%, 60+, 61-)<br>---------------<br>DEL: 203<br>INS: 83	Total count: 5034<br>T: 26 (1%, 25+, 1-)<br>**G: 3096 (62%, 2486+, 610-)**<br>C: 7 (0%, 4+, 3-)<br>**A: 1773 (35%, 1385+, 388-)**<br>N : 132 (3%, 70+, 62-)<br>---------------<br>DEL: 210<br>INS: 73	Total count: 5100<br>T: 69 (1%, 58+, 11-)<br>G: 11 (0%, 6+, 5-)<br>**C: 3237 (63%, 2526+, 711-)**<br>**A: 1655 (32%, 1286+, 369-)**<br>N : 128 (3%, 67+, 61-)<br>---------------<br>DEL: 122<br>INS: 29	Total count: 5135<br>T: 41 (1%, 29+, 12-)<br>**G: 1486 (29%, 1183+, 303-)**<br>C: 17 (0%, 12+, 5-)<br>**A: 3464 (67%, 2637+, 827-)**<br>N : 127 (2%, 70+, 57-)<br>---------------<br>DEL: 46<br>INS: 72	Total count: 5171<br>T: 25 (0%, 22+, 3-)<br>G: 31 (1%, 19+, 12-)<br>C: 16 (0%, 7+, 9-)<br>**A: 4962 (96%, 3727+, 1235-)**<br>N : 137 (3%, 73+, 64-)<br>---------------<br>DEL: 98<br>INS: 35	Total count: 4769<br>T: 11 (0%, 3+, 8-)<br>G: 97 (2%, 5+, 92-)<br>C: 11 (0%, 1+, 10-)<br>**A: 4630 (97%, 1731+, 2899-)**<br>N : 20 (0%, 10+, 10-)<br>---------------<br>DEL: 82<br>INS: 54	Total count: 4577<br>T: 32 (1%, 13+, 19-)<br>G: 8 (0%, 7+, 1-)<br>**C: 4503 (98%, 1613+, 2890-)**<br>A: 13 (0%, 11+, 2-)<br>N : 21 (0%, 11+, 10-)<br>---------------<br>DEL: 254<br>INS: 108	Total count: 4795<br>T: 2 (0%, 0+, 2-)<br>**G: 4726 (99%, 1671+, 3055-)**<br>C: 11 (0%, 9+, 2-)<br>A: 36 (1%, 18+, 18-)<br>N : 20 (0%, 9+, 11-)<br>---------------<br>DEL: 65<br>INS: 11	Total count: 4970<br>T: 19 (0%, 16+, 3-)<br>G: 5 (0%, 1+, 4-)<br>**C: 4892 (98%, 1026+, 3866-)**<br>A: 9 (0%, 0+, 9-)<br>N : 45 (1%, 12+, 33-)<br>---------------<br>DEL: 78<br>INS: 27	Total count: 4961<br>**T: 4898 (99%, 1030+, 3868-)**<br>G: 5 (0%, 1+, 4-)<br>C: 14 (0%, 3+, 11-)<br>A: 3 (0%, 2+, 1-)<br>N : 41 (1%, 9+, 32-)<br>---------------<br>DEL: 96<br>INS: 227	*02/*03/*04
AAL_003	chr1:161.629.989<br>Total count: 6181	chr1:161.629.983<br>Total count: 6404	chr1:161.629.903<br>Total count: 6393	chr1:161.629.864<br>Total count: 6480	chr1:161.629.853<br>Total count: 6521	chr1:161.629.781<br>Total count: 6539	chr1:161.626.282<br>Total count: 6313	chr1:161.626.250<br>Total count: 6052	chr1:161.626.196<br>Total count: 6347	chr1:161.624.609<br>Total count: 6597	chr1:161.624.517<br>Total count: 6578	*03/*04/Novel allele GCGCAA

	T: 82 (1%, 78+, 4-)<br>**G: 3778 (61%, 3118+, 660-)**<br>**C: 2033 (33%, 1637+, 396-)**<br>A: 89 (1%, 45+, 44-)<br>N : 199 (3%, 95+, 104-)<br>---------------<br>DEL: 520<br>INS: 142	**T: 2512 (39%, 2112+, 400-)**<br>G: 95 (1%, 88+, 7-)<br>**C: 3551 (55%, 2735+, 816-)**<br>A: 36 (1%, 31+, 5-)<br>N : 210 (3%, 99+, 111-)<br>---------------<br>DEL: 227<br>INS: 130	T: 32 (1%, 31+, 1-)<br>**G: 3814 (60%, 3086+, 728-)**<br>C: 16 (0%, 10+, 6-)<br>**A: 2307 (36%, 1816+, 491-)**<br>N : 224 (4%, 111+, 113-)<br>---------------<br>DEL: 240<br>INS: 91	T: 68 (1%, 59+, 9-)<br>G: 26 (0%, 16+, 10-)<br>**C: 4213 (65%, 3297+, 916-)**<br>**A: 1944 (30%, 1540+, 404-)**<br>N : 229 (4%, 114+, 115-)<br>---------------<br>DEL: 149<br>INS: 38	T: 34 (1%, 26+, 8-)<br>**G: 1980 (30%, 1576+, 404-)**<br>C: 29 (0%, 22+, 7-)<br>**A: 4247 (65%, 3270+, 977-)**<br>N : 231 (4%, 112+, 119-)<br>---------------<br>DEL: 62<br>INS: 93	T: 20 (0%, 19+, 1-)<br>G: 48 (1%, 37+, 11-)<br>C: 15 (0%, 9+, 6-)<br>**A: 6212 (95%, 4688+, 1524-)**<br>N : 244 (4%, 117+, 127-)<br>---------------<br>DEL: 165<br>INS: 53	T: 7 (0%, 2+, 5-)<br>G: 113 (2%, 7+, 106-)<br>C: 18 (0%, 1+, 17-)<br>**A: 6130 (97%, 2279+, 3851-)**<br>N : 45 (1%, 31+, 14-)<br>---------------<br>DEL: 91<br>INS: 89	T: 40 (1%, 18+, 22-)<br>G: 6 (0%, 3+, 3-)<br>**C: 5957 (98%, 2136+, 3821-)**<br>A: 9 (0%, 8+, 1-)<br>N : 40 (1%, 28+, 12-)<br>---------------<br>DEL: 329<br>INS: 90	T: 7 (0%, 2+, 5-)<br>**G: 6230 (98%, 2192+, 4038-)**<br>C: 12 (0%, 7+, 5-)<br>A: 55 (1%, 27+, 28-)<br>N : 43 (1%, 28+, 15-)<br>---------------<br>DEL: 63<br>INS: 17	T: 26 (0%, 21+, 5-)<br>G: 1 (0%, 0+, 1-)<br>**C: 6499 (99%, 1303+, 5196-)**<br>A: 6 (0%, 0+, 6-)<br>N : 65 (1%, 11+, 54-)<br>---------------<br>DEL: 122<br>INS: 20	**T: 6465 (98%, 1294+, 5171-)**<br>G: 15 (0%, 0+, 15-)<br>C: 29 (0%, 5+, 24-)<br>A: 11 (0%, 5+, 6-)<br>N : 58 (1%, 13+, 45-)<br>---------------<br>DEL: 165<br>INS: 348	
	chr1:161.629.989	chr1:161.629.983	chr1:161.629.903	chr1:161.629.864	chr1:161.629.853	chr1:161.629.781	chr1:161.626.282	chr1:161.626.250	chr1:161.626.196	chr1:161.624.609	chr1:161.624.517	
AAL_004	Total count: 49103<br>T: 896 (2%, 785+, 111-)<br>**G: 21041 (43%, 17619+, 3422-)**<br>**C: 24459 (50%, 19407+, 5052-)**<br>A: 567 (1%, 345+, 222-)<br>N : 2140 (4%, 1113+, 1027-)<br>---------------<br>DEL: 4151<br>INS: 1143	Total count: 50879<br>**T: 28259 (56%, 23453+, 4806-)**<br>G: 798 (2%, 664+, 134-)<br>**C: 19090 (38%, 14782+, 4308-)**<br>A: 417 (1%, 205+, 212-)<br>N : 2315 (5%, 1161+, 1154-)<br>---------------<br>DEL: 2176<br>INS: 1055	Total count: 51788<br>T: 226 (0%, 220+, 6-)<br>**G: 23728 (46%, 19421+, 4307-)**<br>C: 135 (0%, 96+, 39-)<br>**A: 25217 (49%, 19895+, 5322-)**<br>N : 2482 (5%, 1287+, 1195-)<br>---------------<br>DEL: 1602<br>INS: 881	Total count: 52199<br>T: 809 (2%, 731+, 78-)<br>G: 29 (0%, 21+, 8-)<br>**C: 48606 (93%, 38385+, 10221-)**<br>A: 262 (1%, 242+, 20-)<br>N : 2493 (5%, 1273+, 1220-)<br>---------------<br>DEL: 1247<br>INS: 213	Total count: 52505<br>T: 532 (1%, 402+, 130-)<br>**G: 46025 (88%, 36499+, 9526-)**<br>C: 258 (0%, 220+, 38-)<br>A: 3166 (6%, 2403+, 763-)<br>N : 2524 (5%, 1286+, 1238-)<br>---------------<br>DEL: 909<br>INS: 2027	Total count: 52815<br>T: 205 (0%, 196+, 9-)<br>**G: 23512 (45%, 17903+, 5609-)**<br>C: 195 (0%, 121+, 74-)<br>**A: 26164 (50%, 19212+, 6952-)**<br>N : 2739 (5%, 1396+, 1343-)<br>---------------<br>DEL: 934<br>INS: 690	Total count: 53567<br>T: 101 (0%, 24+, 77-)<br>G: 947 (2%, 54+, 893-)<br>C: 159 (0%, 11+, 148-)<br>**A: 52208 (97%, 19601+, 32607-)**<br>N : 152 (0%, 75+, 77-)<br>---------------<br>DEL: 754<br>INS: 996	Total count: 50817<br>T: 377 (1%, 196+, 181-)<br>G: 104 (0%, 71+, 33-)<br>**C: 50052 (98%, 17978+, 32074-)**<br>A: 134 (0%, 103+, 31-)<br>N : 150 (0%, 75+, 75-)<br>---------------<br>DEL: 3474<br>INS: 1101	Total count: 53396<br>T: 54 (0%, 12+, 42-)<br>**G: 52569 (98%, 19228+, 33341-)**<br>C: 146 (0%, 92+, 54-)<br>A: 475 (1%, 127+, 348-)<br>N : 152 (0%, 78+, 74-)<br>---------------<br>DEL: 857<br>INS: 199	Total count: 52605<br>T: 173 (0%, 158+, 15-)<br>G: 26 (0%, 1+, 25-)<br>**C: 52037 (99%, 10293+, 41744-)**<br>A: 97 (0%, 1+, 96-)<br>N : 272 (1%, 42+, 230-)<br>---------------<br>DEL: 872<br>INS: 256	Total count: 52218<br>**T: 51645 (99%, 10277+, 41368-)**<br>G: 102 (0%, 16+, 86-)<br>C: 179 (0%, 31+, 148-)<br>A: 70 (0%, 36+, 34-)<br>N : 222 (0%, 35+, 187-)<br>---------------<br>DEL: 1147<br>INS: 2666	_*01/*05_<br>*has SNP rs368410676<br><br>chr1:161.629.800<br><br>**T: 20961 (42%, 15664+, 5297-)**<br>**G: 23678 (48%, 18859+, 4819-)**
	chr1:161.629.989	chr1:161.629.983	chr1:161.629.903	chr1:161.629.864	chr1:161.629.853	chr1:161.629.781	chr1:161.626.282	chr1:161.626.250	chr1:161.626.196	chr1:161.624.609	chr1:161.624.517	
AAL_005	Total count: 46279<br>T: 1361 (3%, 1154+, 207-)<br>G: 283 (1%, 252+, 31-)<br>**C: 43931 (95%, 33720+, 10211-)**<br>A: 414 (1%, 384+, 30-)<br>N : 290 (1%, 188+, 102-)<br>---------------<br>DEL: 2227<br>INS: 995	Total count: 46179<br>**T: 44212 (96%, 34807+, 9405-)**<br>G: 891 (2%, 634+, 257-)<br>C: 219 (0%, 111+, 108-)<br>A: 575 (1%, 162+, 413-)<br>N : 282 (1%, 185+, 97-)<br>---------------<br>DEL: 2322<br>INS: 669	Total count: 47184<br>T: 210 (0%, 206+, 4-)<br>**G: 22162 (47%, 18211+, 3951-)**<br>C: 128 (0%, 88+, 40-)<br>**A: 24395 (52%, 18150+, 6245-)**<br>N : 289 (1%, 190+, 99-)<br>---------------<br>DEL: 1335<br>INS: 770	Total count: 47390<br>T: 665 (1%, 558+, 107-)<br>G: 32 (0%, 20+, 12-)<br>**C: 46148 (97%, 35476+, 10672-)**<br>A: 232 (0%, 215+, 17-)<br>N : 313 (1%, 207+, 106-)<br>---------------<br>DEL: 1188<br>INS: 199	Total count: 48376<br>T: 195 (0%, 118+, 77-)<br>G: 333 (1%, 253+, 80-)<br>C: 204 (0%, 161+, 43-)<br>**A: 47329 (98%, 36384+, 10945-)**<br>N : 315 (1%, 207+, 108-)<br>---------------<br>DEL: 169<br>INS: 249	Total count: 47524<br>T: 284 (1%, 279+, 5-)<br>G: 457 (1%, 305+, 152-)<br>C: 234 (0%, 147+, 87-)<br>**A: 46237 (97%, 34838+, 11399-)**<br>N : 312 (1%, 195+, 117-)<br>---------------<br>DEL: 1320<br>INS: 371	Total count: 46872<br>T: 87 (0%, 24+, 63-)<br>G: 768 (2%, 59+, 709-)<br>C: 132 (0%, 13+, 119-)<br>**A: 45724 (98%, 19254+, 26470-)**<br>N : 161 (0%, 73+, 88-)<br>---------------<br>DEL: 654<br>INS: 794	Total count: 44480<br>T: 357 (1%, 197+, 160-)<br>G: 114 (0%, 92+, 22-)<br>**C: 43719 (98%, 17660+, 26059-)**<br>A: 126 (0%, 103+, 23-)<br>N : 164 (0%, 72+, 92-)<br>---------------<br>DEL: 3028<br>INS: 946	Total count: 46780<br>T: 51 (0%, 13+, 38-)<br>**G: 46045 (98%, 18963+, 27082-)**<br>C: 113 (0%, 74+, 39-)<br>A: 413 (1%, 137+, 276-)<br>N : 158 (0%, 71+, 87-)<br>---------------<br>DEL: 716<br>INS: 177	Total count: 46136<br>T: 209 (0%, 191+, 18-)<br>G: 33 (0%, 7+, 26-)<br>**C: 45353 (98%, 10425+, 34928-)**<br>A: 88 (0%, 1+, 87-)<br>N : 453 (1%, 43+, 410-)<br>---------------<br>DEL: 1013<br>INS: 229	Total count: 46069<br>**T: 45383 (99%, 10726+, 34657-)**<br>G: 64 (0%, 17+, 47-)<br>C: 151 (0%, 35+, 116-)<br>A: 93 (0%, 42+, 51-)<br>N : 378 (1%, 40+, 338-)<br>---------------<br>DEL: 1049<br>INS: 2263	_*02/*02 var 194 A>G_

	chr1:161.629.989	chr1:161.629.983	chr1:161.629.903	chr1:161.629.864	chr1:161.629.853	chr1:161.629.781	chr1:161.626.282	chr1:161.626.250	chr1:161.626.196	chr1:161.624.609	chr1:161.624.517	
AAL_006	Total count: 25241<br>T: 534 (2%, 454+, 80-)<br>**G: 7083 (28%, 5865+, 1218-)**<br>**C: 16284 (65%, 12660+, 3624-)**<br>A: 289 (1%, 193+, 96-)<br>N : 1051 (4%, 489+, 562-)<br>---------------<br>DEL: 1818<br>INS: 554	Total count: 25858<br>**T: 17532 (68%, 14100+, 3432-)**<br>G: 393 (2%, 313+, 80-)<br>**C: 6566 (25%, 4939+, 1627-)**<br>A: 225 (1%, 91+, 134-)<br>N : 1142 (4%, 523+, 619-)<br>---------------<br>DEL: 1081<br>INS: 515	Total count: 26065<br>T: 121 (0%, 118+, 3-)<br>**G: 15804 (61%, 12648+, 3156-)**<br>C: 58 (0%, 44+, 14-)<br>**A: 8829 (34%, 6667+, 2162-)**<br>N : 1253 (5%, 594+, 659-)<br>---------------<br>DEL: 973<br>INS: 490	Total count: 26442<br>T: 226 (1%, 177+, 49-)<br>G: 88 (0%, 40+, 48-)<br>**C: 16667 (63%, 12867+, 3800-)**<br>**A: 8199 (31%, 6375+, 1824-)**<br>N : 1262 (5%, 596+, 666-)<br>---------------<br>DEL: 608<br>INS: 199	Total count: 26701<br>T: 156 (1%, 115+, 41-)<br>**G: 7452 (28%, 5725+, 1727-)**<br>C: 129 (0%, 111+, 18-)<br>**A: 17688 (66%, 13441+, 4247-)**<br>N : 1276 (5%, 592+, 684-)<br>---------------<br>DEL: 219<br>INS: 397	Total count: 26467<br>T: 130 (0%, 127+, 3-)<br>G: 253 (1%, 176+, 77-)<br>C: 130 (0%, 88+, 42-)<br>**A: 24629 (93%, 18403+, 6226-)**<br>N : 1325 (5%, 614+, 711-)<br>---------------<br>DEL: 699<br>INS: 193	Total count: 25167<br>T: 53 (0%, 13+, 40-)<br>G: 457 (2%, 38+, 419-)<br>C: 69 (0%, 4+, 65-)<br>**A: 24510 (97%, 9696+, 14814-)**<br>N : 78 (0%, 35+, 43-)<br>---------------<br>DEL: 349<br>INS: 484	Total count: 23753<br>T: 163 (1%, 90+, 73-)<br>G: 54 (0%, 39+, 15-)<br>**C: 23401 (99%, 8918+, 14483-)**<br>A: 68 (0%, 53+, 15-)<br>N : 67 (0%, 32+, 35-)<br>---------------<br>DEL: 1638<br>INS: 523	Total count: 25066<br>T: 34 (0%, 11+, 23-)<br>**G: 24626 (98%, 9386+, 15240-)**<br>C: 76 (0%, 49+, 27-)<br>A: 254 (1%, 68+, 186-)<br>N : 76 (0%, 39+, 37-)<br>---------------<br>DEL: 399<br>INS: 85	Total count: 23986<br>T: 97 (0%, 89+, 8-)<br>G: 13 (0%, 0+, 13-)<br>**C: 23725 (99%, 5430+, 18295-)**<br>A: 45 (0%, 1+, 44-)<br>N : 106 (0%, 16+, 90-)<br>---------------<br>DEL: 440<br>INS: 111	Total count: 23854<br>**T: 23624 (99%, 5371+, 18253-)**<br>G: 35 (0%, 10+, 25-)<br>C: 71 (0%, 16+, 55-)<br>A: 33 (0%, 13+, 20-)<br>N : 91 (0%, 13+, 78-)<br>---------------<br>DEL: 466<br>INS: 1137	*02/*03/*04
	chr1:161.629.989	chr1:161.629.983	chr1:161.629.903	chr1:161.629.864	chr1:161.629.853	chr1:161.629.781	chr1:161.626.282	chr1:161.626.250	chr1:161.626.196	chr1:161.624.609	chr1:161.624.517	
AAL_007	Total count: 960<br>T: 16 (2%, 14+, 2-)<br>**G: 389 (41%, 317+, 72-)**<br>**C: 489 (51%, 363+, 126-)**<br>A: 20 (2%, 12+, 8-)<br>N : 46 (5%, 27+, 19-)<br>---------------<br>DEL: 83<br>INS: 25	Total count: 998<br>**T: 565 (57%, 446+, 119-)**<br>G: 14 (1%, 9+, 5-)<br>**C: 366 (37%, 274+, 92-)**<br>A: 6 (1%, 2+, 4-)<br>N : 47 (5%, 28+, 19-)<br>---------------<br>DEL: 41<br>INS: 22	Total count: 1004<br>T: 6 (1%, 6+, 0-)<br>**G: 472 (47%, 374+, 98-)**<br>C: 4 (0%, 4+, 0-)<br>**A: 473 (47%, 360+, 113-)**<br>N : 49 (5%, 31+, 18-)<br>---------------<br>DEL: 29<br>INS: 21	Total count: 1008<br>T: 6 (1%, 4+, 2-)<br>G: 2 (0%, 2+, 0-)<br>**C: 939 (93%, 712+, 227-)**<br>A: 11 (1%, 8+, 3-)<br>N : 50 (5%, 31+, 19-)<br>---------------<br>DEL: 23<br>INS: 3	Total count: 1011<br>T: 13 (1%, 10+, 3-)<br>**G: 845 (84%, 644+, 201-)**<br>C: 11 (1%, 11+, 0-)<br>A: 90 (9%, 69+, 21-)<br>N : 52 (5%, 32+, 20-)<br>---------------<br>DEL: 19<br>INS: 58	Total count: 1071<br>T: 3 (0%, 3+, 0-)<br>G: 14 (1%, 6+, 8-)<br>C: 7 (1%, 2+, 5-)<br>**A: 992 (93%, 702+, 290-)**<br>N : 55 (5%, 29+, 26-)<br>---------------<br>DEL: 21<br>INS: 8	Total count: 1223<br>T: 1 (0%, 0+, 1-)<br>G: 24 (2%, 0+, 24-)<br>C: 1 (0%, 0+, 1-)<br>**A: 1187 (97%, 411+, 776-)**<br>N : 10 (1%, 3+, 7-)<br>---------------<br>DEL: 16<br>INS: 24	Total count: 1153<br>T: 7 (1%, 2+, 5-)<br>G: 0<br>**C: 1137 (99%, 378+, 759-)**<br>A: 1 (0%, 1+, 0-)<br>N : 8 (1%, 3+, 5-)<br>---------------<br>DEL: 82<br>INS: 28	Total count: 1221<br>T: 1 (0%, 0+, 1-)<br>**G: 1189 (97%, 393+, 796-)**<br>C: 3 (0%, 2+, 1-)<br>A: 20 (2%, 5+, 15-)<br>N : 8 (1%, 4+, 4-)<br>---------------<br>DEL: 18<br>INS: 5	Total count: 1203<br>T: 2 (0%, 2+, 0-)<br>G: 0<br>**C: 1189 (99%, 223+, 966-)**<br>A: 4 (0%, 0+, 4-)<br>N : 8 (1%, 1+, 7-)<br>---------------<br>DEL: 22<br>INS: 11	Total count: 1196<br>**T: 1181 (99%, 220+, 961-)**<br>G: 2 (0%, 0+, 2-)<br>C: 4 (0%, 0+, 4-)<br>A: 1 (0%, 0+, 1-)<br>N : 8 (1%, 1+, 7-)<br>---------------<br>DEL: 29<br>INS: 71	*04/*05<br>*has SNP rs368410676 chr1:161.629.800<br>Total count: 1027<br>**T : 408 (40%, 282+, 126-)**<br>**G: 494 (48%, 368+, 126-)**
	chr1:161.629.989	chr1:161.629.983	chr1:161.629.903	chr1:161.629.864	chr1:161.629.853	chr1:161.629.781	chr1:161.626.282	chr1:161.626.250	chr1:161.626.196	chr1:161.624.609	chr1:161.624.517	
AAL_008	Total count: 748<br>T: 19 (3%, 17+, 2-)<br>G: 27 (4%, 21+, 6-)<br>**C: 686 (92%, 551+, 135-)**<br>A: 10 (1%, 9+, 1-)<br>N : 6 (1%, 4+, 2-)<br>---------------<br>DEL: 46	Total count: 755<br>**T: 700 (93%, 570+, 130-)**<br>G: 22 (3%, 20+, 2-)<br>C: 23 (3%, 16+, 7-)<br>A: 5 (1%, 1+, 4-)<br>N : 5 (1%, 4+, 1-)<br>---------------<br>DEL: 39	Total count: 743<br>T: 4 (1%, 4+, 0-)<br>**G: 679 (91%, 564+, 115-)**<br>C: 1 (0%, 1+, 0-)<br>A: 53 (7%, 34+, 19-)<br>N : 6 (1%, 4+, 2-)<br>---------------<br>DEL: 46	Total count: 767<br>T: 5 (1%, 2+, 3-)<br>G: 9 (1%, 5+, 4-)<br>**C: 376 (49%, 292+, 84-)**<br>**A: 369 (48%, 301+, 68-)**<br>N : 8 (1%, 5+, 3-)<br>---------------<br>DEL: 24	Total count: 793<br>T: 1 (0%, 0+, 1-)<br>G: 36 (5%, 28+, 8-)<br>C: 7 (1%, 6+, 1-)<br>**A: 740 (93%, 585+, 155-)**<br>N : 9 (1%, 6+, 3-)<br>---------------<br>DEL: 3	Total count: 773<br>T: 5 (1%, 5+, 0-)<br>G: 19 (2%, 12+, 7-)<br>C: 1 (0%, 1+, 0-)<br>**A: 740 (96%, 566+, 174-)**<br>N : 8 (1%, 6+, 2-)<br>---------------<br>DEL: 20	Total count: 775<br>T: 0<br>G: 9 (1%, 0+, 9-)<br>C: 1 (0%, 0+, 1-)<br>**A: 759 (98%, 269+, 490-)**<br>N : 6 (1%, 4+, 2-)<br>---------------<br>DEL: 9<br>INS: 15	Total count: 733<br>T: 5 (1%, 2+, 3-)<br>G: 2 (0%, 2+, 0-)<br>**C: 718 (98%, 241+, 477-)**<br>A: 1 (0%, 1+, 0-)<br>N : 7 (1%, 4+, 3-)<br>---------------<br>DEL: 48	Total count: 772<br>T: 1 (0%, 1+, 0-)<br>**G: 757 (98%, 262+, 495-)**<br>C: 1 (0%, 1+, 0-)<br>A: 8 (1%, 3+, 5-)<br>N : 5 (1%, 3+, 2-)<br>---------------<br>DEL: 8	Total count: 789<br>T: 1 (0%, 1+, 0-)<br>G: 0<br>**C: 780 (99%, 141+, 639-)**<br>A: 1 (0%, 0+, 1-)<br>N : 7 (1%, 1+, 6-)<br>---------------<br>DEL: 15<br>INS: 3	Total count: 786<br>**T: 772 (98%, 136+, 636-)**<br>G: 1 (0%, 0+, 1-)<br>C: 7 (1%, 1+, 6-)<br>A: 1 (0%, 0+, 1-)<br>N : 5 (1%, 1+, 4-)<br>---------------<br>DEL: 14	*02/*03

	INS: 20	INS: 12	INS: 20	INS: 9	INS: 10	INS: 10		INS: 20	INS: 2		INS: 45	
AAL_009	chr1:161.629.989<br>Total count: 16645<br>T: 438 (3%, 387+, 51-)<br>G: 90 (1%, 86+, 4-)<br>**C: 15813 (95%, 12504+, 3309-)**<br>A: 167 (1%, 158+, 9-)<br>N : 137 (1%, 75+, 62-)<br>---------------<br>DEL: 794<br>INS: 320	chr1:161.629.983<br>Total count: 16634<br>**T: 15942 (96%, 12891+, 3051-)**<br>G: 304 (2%, 212+, 92-)<br>C: 78 (0%, 49+, 29-)<br>A: 172 (1%, 63+, 109-)<br>N : 138 (1%, 75+, 63-)<br>---------------<br>DEL: 801<br>INS: 246	chr1:161.629.903<br>Total count: 16713<br>T: 102 (1%, 98+, 4-)<br>**G: 15830 (95%, 12993+, 2837-)**<br>C: 48 (0%, 35+, 13-)<br>A: 587 (4%, 274+, 313-)<br>N : 146 (1%, 79+, 67-)<br>---------------<br>DEL: 757<br>INS: 351	chr1:161.629.864<br>Total count: 17077<br>T: 300 (2%, 269+, 31-)<br>G: 6 (0%, 4+, 2-)<br>**C: 16535 (97%, 13048+, 3487-)**<br>A: 78 (0%, 74+, 4-)<br>N : 158 (1%, 81+, 77-)<br>---------------<br>DEL: 421<br>INS: 60	chr1:161.629.853<br>Total count: 17246<br>T: 128 (1%, 94+, 34-)<br>**G: 7853 (46%, 6175+, 1678-)**<br>C: 91 (1%, 80+, 11-)<br>**A: 9016 (52%, 7064+, 1952-)**<br>N : 158 (1%, 80+, 78-)<br>---------------<br>DEL: 187<br>INS: 380	chr1:161.629.781<br>Total count: 17235<br>T: 97 (1%, 94+, 3-)<br>G: 176 (1%, 108+, 68-)<br>C: 87 (1%, 59+, 28-)<br>**A: 16692 (97%, 12034+, 4658-)**<br>N : 183 (1%, 84+, 99-)<br>---------------<br>DEL: 429<br>INS: 146	chr1:161.626.282<br>Total count: 17523<br>T: 33 (0%, 10+, 23-)<br>G: 323 (2%, 15+, 308-)<br>C: 40 (0%, 1+, 39-)<br>**A: 17073 (97%, 6587+, 10486-)**<br>N : 54 (0%, 29+, 25-)<br>---------------<br>DEL: 213<br>INS: 311	chr1:161.626.250<br>Total count: 16598<br>T: 104 (1%, 54+, 50-)<br>G: 48 (0%, 36+, 12-)<br>**C: 16368 (99%, 6050+, 10318-)**<br>A: 33 (0%, 28+, 5-)<br>N : 45 (0%, 23+, 22-)<br>---------------<br>DEL: 1108<br>INS: 354	chr1:161.626.196<br>Total count: 17456<br>T: 18 (0%, 2+, 16-)<br>**G: 17168 (98%, 6463+, 10705-)**<br>C: 46 (0%, 28+, 18-)<br>A: 166 (1%, 52+, 114-)<br>N : 58 (0%, 27+, 31-)<br>---------------<br>DEL: 258<br>INS: 55	chr1:161.624.609<br>Total count: 16982<br>T: 76 (0%, 65+, 11-)<br>G: 12 (0%, 2+, 10-)<br>**C: 16806 (99%, 3273+, 13533-)**<br>A: 25 (0%, 0+, 25-)<br>N : 63 (0%, 5+, 58-)<br>---------------<br>DEL: 331<br>INS: 71	chr1:161.624.517<br>Total count: 16946<br>**T: 16786 (99%, 3329+, 13457-)**<br>G: 34 (0%, 5+, 29-)<br>C: 43 (0%, 3+, 40-)<br>A: 27 (0%, 13+, 14-)<br>N : 56 (0%, 5+, 51-)<br>---------------<br>DEL: 343<br>INS: 915	*02/*05<br>*has SNP rs368410676 chr1:161.629.800<br><br>Total count: 16247<br>**T: 6852 (42%, 5060+, 1792-)**<br>**G: 8263 (51%, 6415+, 1848-)**
AAL_010	chr1:161.629.989<br>Total count: 34504<br>T: 969 (3%, 833+, 136-)<br>G: 206 (1%, 188+, 18-)<br>**C: 32666 (95%, 25679+, 6987-)**<br>A: 307 (1%, 287+, 20-)<br>N : 356 (1%, 206+, 150-)<br>---------------<br>DEL: 1680<br>INS: 697	chr1:161.629.983<br>Total count: 34576<br>**T: 33018 (95%, 26541+, 6477-)**<br>G: 661 (2%, 475+, 186-)<br>C: 177 (1%, 90+, 87-)<br>A: 368 (1%, 109+, 259-)<br>N : 352 (1%, 207+, 145-)<br>---------------<br>DEL: 1612<br>INS: 504	chr1:161.629.903<br>Total count: 34441<br>T: 192 (1%, 191+, 1-)<br>**G: 32605 (95%, 26669+, 5936-)**<br>C: 117 (0%, 87+, 30-)<br>A: 1170 (3%, 523+, 647-)<br>N : 357 (1%, 211+, 146-)<br>---------------<br>DEL: 1787<br>INS: 717	chr1:161.629.864<br>Total count: 35310<br>T: 645 (2%, 571+, 74-)<br>G: 26 (0%, 19+, 7-)<br>**C: 34107 (97%, 26769+, 7338-)**<br>A: 156 (0%, 148+, 8-)<br>N : 376 (1%, 217+, 159-)<br>---------------<br>DEL: 946<br>INS: 139	chr1:161.629.853<br>Total count: 35761<br>T: 286 (1%, 204+, 82-)<br>**G: 16109 (45%, 12621+, 3488-)**<br>C: 202 (1%, 176+, 26-)<br>**A: 18786 (53%, 14581+, 4205-)**<br>N : 378 (1%, 218+, 160-)<br>---------------<br>DEL: 386<br>INS: 797	chr1:161.629.781<br>Total count: 36116<br>T: 203 (1%, 200+, 3-)<br>G: 349 (1%, 228+, 121-)<br>C: 179 (0%, 119+, 60-)<br>**A: 34961 (97%, 24875+, 10086-)**<br>N : 424 (1%, 218+, 206-)<br>---------------<br>DEL: 938<br>INS: 248	chr1:161.626.282<br>Total count: 37147<br>T: 60 (0%, 18+, 42-)<br>G: 721 (2%, 49+, 672-)<br>C: 113 (0%, 6+, 107-)<br>**A: 36113 (97%, 13875+, 22238-)**<br>N : 140 (0%, 62+, 78-)<br>---------------<br>DEL: 490<br>INS: 700	chr1:161.626.250<br>Total count: 35142<br>T: 268 (1%, 147+, 121-)<br>G: 77 (0%, 55+, 22-)<br>**C: 34590 (98%, 12682+, 21908-)**<br>A: 78 (0%, 60+, 18-)<br>N : 129 (0%, 50+, 79-)<br>---------------<br>DEL: 2480<br>INS: 744	chr1:161.626.196<br>Total count: 37061<br>T: 31 (0%, 6+, 25-)<br>**G: 36476 (98%, 13633+, 22843-)**<br>C: 103 (0%, 66+, 37-)<br>A: 330 (1%, 85+, 245-)<br>N : 121 (0%, 52+, 69-)<br>---------------<br>DEL: 567<br>INS: 143	chr1:161.624.609<br>Total count: 36515<br>T: 138 (0%, 112+, 26-)<br>G: 23 (0%, 5+, 18-)<br>**C: 36091 (99%, 7108+, 28983-)**<br>A: 74 (0%, 0+, 74-)<br>N : 189 (1%, 30+, 159-)<br>---------------<br>DEL: 694<br>INS: 179	chr1:161.624.517<br>Total count: 36324<br>**T: 35921 (99%, 7170+, 28751-)**<br>G: 56 (0%, 9+, 47-)<br>C: 117 (0%, 18+, 99-)<br>A: 59 (0%, 29+, 30-)<br>N : 171 (0%, 24+, 147-)<br>---------------<br>DEL: 832<br>INS: 1922	*02/*05<br>*has SNP rs368410676 chr1:161.629.800<br><br>Total count: 33698<br>**T: 14036 (42%, 10301+, 3735-)**<br>**G: 17280 (51%, 13230+, 4050-)**
AAL_011	chr1:161.629.989<br>Total count: 51825<br>T: 984 (2%, 850+, 134-)<br>**G: 21552 (42%, 18106+, 3446-)**<br>**C: 27066 (52%, 21452+, 5614-)**<br>A: 638 (1%,	chr1:161.629.983<br>Total count: 53559<br>**T: 30872 (58%, 25526+, 5346-)**<br>G: 747 (1%, 618+, 129-)<br>**C: 19747 (37%, 15310+, 4437-)**<br>A: 465 (1%,	chr1:161.629.903<br>Total count: 54652<br>T: 247 (0%, 240+, 7-)<br>**G: 26422 (48%, 21544+, 4878-)**<br>C: 139 (0%, 104+, 35-)<br>**A: 25980 (48%, 20498+,**	chr1:161.629.864<br>Total count: 54967<br>T: 697 (1%, 592+, 105-)<br>G: 40 (0%, 30+, 10-)<br>**C: 52103 (95%, 41197+, 10906-)**<br>A: 248 (0%, 227+, 21-)	chr1:161.629.853<br>Total count: 55487<br>T: 379 (1%, 280+, 99-)<br>**G: 22905 (41%, 18172+, 4733-)**<br>C: 239 (0%, 197+, 42-)<br>**A: 30070 (54%, 23291+,**	chr1:161.629.781<br>Total count: 55671<br>T: 223 (0%, 215+, 8-)<br>**G: 24028 (43%, 18759+, 5269-)**<br>C: 232 (0%, 156+, 76-)<br>**A: 29232 (53%, 22002+,**	chr1:161.626.282<br>Total count: 57411<br>T: 100 (0%, 32+, 68-)<br>G: 1115 (2%, 83+, 1032-)<br>C: 146 (0%, 16+, 130-)<br>**A: 55792 (97%, 20887+, 34905-)**	chr1:161.626.250<br>Total count: 54360<br>T: 402 (1%, 213+, 189-)<br>G: 115 (0%, 92+, 23-)<br>**C: 53470 (98%, 19122+, 34348-)**<br>A: 138 (0%, 114+, 24-)	chr1:161.626.196<br>Total count: 57268<br>T: 71 (0%, 30+, 41-)<br>**G: 56272 (98%, 20493+, 35779-)**<br>C: 160 (0%, 101+, 59-)<br>A: 516 (1%, 148+, 368-)	chr1:161.624.609<br>Total count: 55944<br>T: 264 (0%, 221+, 43-)<br>G: 33 (0%, 4+, 29-)<br>**C: 55217 (99%, 11602+, 43615-)**<br>A: 99 (0%, 6+, 93-)	chr1:161.624.517<br>Total count: 55646<br>**T: 54944 (99%, 11708+, 43236-)**<br>G: 93 (0%, 19+, 74-)<br>C: 231 (0%, 52+, 179-)<br>A: 79 (0%, 35+, 44-)	*01/*02

	386+, 252-)<br>N : 1585 (3%, 733+, 852-)<br>---------------<br>DEL: 4211<br>INS: 1130	212+, 253-)<br>N : 1728 (3%, 759+, 969-)<br>---------------<br>DEL: 2276<br>INS: 1201	**5482-)**<br>N : 1864 (3%, 859+, 1005-)<br>---------------<br>DEL: 1602<br>INS: 1017	N : 1879 (3%, 861+, 1018-)<br>---------------<br>DEL: 1314<br>INS: 226	**6779-)**<br>N : 1894 (3%, 863+, 1031-)<br>---------------<br>DEL: 585<br>INS: 1135	**7230-)**<br>N : 1956 (4%, 885+, 1071-)<br>---------------<br>DEL: 1017<br>INS: 734	N : 258 (0%, 139+, 119-)<br>---------------<br>DEL: 797<br>INS: 1191	N : 235 (0%, 119+, 116-)<br>---------------<br>DEL: 3839<br>INS: 1166	N : 249 (0%, 130+, 119-)<br>---------------<br>DEL: 891<br>INS: 185	N : 331 (1%, 47+, 284-)<br>---------------<br>DEL: 1078<br>INS: 221	N : 299 (1%, 46+, 253-)<br>---------------<br>DEL: 1304<br>INS: 2924	
AAL_012	chr1:161.629.989<br>Total count: 34469<br>T: 750 (2%, 630+, 120-)<br>**G: 10133 (29%, 8316+, 1817-)**<br>**C: 21264 (62%, 16928+, 4336-)**<br>A: 380 (1%, 232+, 148-)<br>N : 1942 (6%, 1214+, 728-)<br>---------------<br>DEL: 2636<br>INS: 758	chr1:161.629.983<br>Total count: 35514<br>**T: 23165 (65%, 19015+, 4150-)**<br>G: 538 (2%, 404+, 134-)<br>**C: 9407 (26%, 7017+, 2390-)**<br>A: 298 (1%, 136+, 162-)<br>N : 2106 (6%, 1263+, 843-)<br>---------------<br>DEL: 1502<br>INS: 702	chr1:161.629.903<br>Total count: 35867<br>T: 166 (0%, 159+, 7-)<br>**G: 21157 (59%, 17262+, 3895-)**<br>C: 95 (0%, 70+, 25-)<br>**A: 12262 (34%, 9344+, 2918-)**<br>N : 2187 (6%, 1331+, 856-)<br>---------------<br>DEL: 1368<br>INS: 712	chr1:161.629.864<br>Total count: 36335<br>T: 387 (1%, 316+, 71-)<br>G: 126 (0%, 74+, 52-)<br>**C: 22803 (63%, 17747+, 5056-)**<br>**A: 10816 (30%, 8601+, 2215-)**<br>N : 2203 (6%, 1328+, 875-)<br>---------------<br>DEL: 856<br>INS: 287	chr1:161.629.853<br>Total count: 36717<br>T: 208 (1%, 141+, 67-)<br>**G: 10721 (29%, 8248+, 2473-)**<br>C: 173 (0%, 146+, 27-)<br>**A: 23390 (64%, 18148+, 5242-)**<br>N : 2225 (6%, 1323+, 902-)<br>---------------<br>DEL: 327<br>INS: 590	chr1:161.629.781<br>Total count: 36330<br>T: 154 (0%, 151+, 3-)<br>G: 308 (1%, 211+, 97-)<br>C: 168 (0%, 107+, 61-)<br>**A: 33435 (92%, 25368+, 8067-)**<br>N : 2265 (6%, 1324+, 941-)<br>---------------<br>DEL: 1037<br>INS: 244	chr1:161.626.282<br>Total count: 36893<br>T: 66 (0%, 22+, 44-)<br>G: 824 (2%, 94+, 730-)<br>C: 82 (0%, 7+, 75-)<br>**A: 34902 (95%, 13224+, 21678-)**<br>N : 1019 (3%, 407+, 612-)<br>---------------<br>DEL: 635<br>INS: 734	chr1:161.626.250<br>Total count: 34925<br>T: 351 (1%, 161+, 190-)<br>G: 73 (0%, 44+, 29-)<br>**C: 33437 (96%, 12140+, 21297-)**<br>A: 91 (0%, 71+, 20-)<br>N : 973 (3%, 357+, 616-)<br>---------------<br>DEL: 2493<br>INS: 762	chr1:161.626.196<br>Total count: 36886<br>T: 35 (0%, 13+, 22-)<br>**G: 35325 (96%, 12825+, 22500-)**<br>C: 102 (0%, 65+, 37-)<br>A: 347 (1%, 103+, 244-)<br>N : 1077 (3%, 412+, 665-)<br>---------------<br>DEL: 615<br>INS: 125	chr1:161.624.609<br>Total count: 36234<br>T: 446 (1%, 268+, 178-)<br>G: 12 (0%, 2+, 10-)<br>**C: 34549 (95%, 7059+, 27490-)**<br>A: 77 (0%, 1+, 76-)<br>N : 1150 (3%, 235+, 915-)<br>---------------<br>DEL: 668<br>INS: 164	chr1:161.624.517<br>Total count: 36004<br>**T: 34356 (95%, 7051+, 27305-)**<br>G: 67 (0%, 13+, 54-)<br>C: 402 (1%, 161+, 241-)<br>A: 62 (0%, 27+, 35-)<br>N : 1117 (3%, 232+, 885-)<br>---------------<br>DEL: 847<br>INS: 1851	**02/*03/*04*
AAL_013	chr1:161.629.989<br>Total count: 10508<br>T: 202 (2%, 178+, 24-)<br>**G: 4738 (45%, 3982+, 756-)**<br>**C: 5316 (51%, 4359+, 957-)**<br>A: 134 (1%, 79+, 55-)<br>N : 118 (1%, 62+, 56-)<br>---------------<br>DEL: 887<br>INS: 202	chr1:161.629.983<br>Total count: 10901<br>**T: 6223 (57%, 5288+, 935-)**<br>G: 157 (1%, 134+, 23-)<br>**C: 4320 (40%, 3362+, 958-)**<br>A: 76 (1%, 32+, 44-)<br>N : 125 (1%, 64+, 61-)<br>---------------<br>DEL: 436<br>INS: 241	chr1:161.629.903<br>Total count: 10820<br>T: 54 (0%, 53+, 1-)<br>**G: 10227 (95%, 8489+, 1738-)**<br>C: 26 (0%, 21+, 5-)<br>A: 369 (3%, 165+, 204-)<br>N : 144 (1%, 76+, 68-)<br>---------------<br>DEL: 560<br>INS: 226	chr1:161.629.864<br>Total count: 11107<br>T: 167 (2%, 148+, 19-)<br>G: 8 (0%, 7+, 1-)<br>**C: 10723 (97%, 8527+, 2196-)**<br>A: 64 (1%, 60+, 4-)<br>N : 145 (1%, 75+, 70-)<br>---------------<br>DEL: 266<br>INS: 49	chr1:161.629.853<br>Total count: 11320<br>T: 48 (0%, 31+, 17-)<br>G: 103 (1%, 79+, 24-)<br>C: 51 (0%, 41+, 10-)<br>**A: 10971 (97%, 8729+, 2242-)**<br>N : 147 (1%, 75+, 72-)<br>---------------<br>DEL: 38<br>INS: 56	chr1:161.629.781<br>Total count: 11073<br>T: 56 (1%, 54+, 2-)<br>G: 117 (1%, 75+, 42-)<br>C: 52 (0%, 34+, 18-)<br>**A: 10701 (97%, 8342+, 2359-)**<br>N : 147 (1%, 72+, 75-)<br>---------------<br>DEL: 291<br>INS: 81	chr1:161.626.282<br>Total count: 10924<br>T: 26 (0%, 6+, 20-)<br>G: 207 (2%, 16+, 191-)<br>C: 40 (0%, 3+, 37-)<br>**A: 10617 (97%, 4064+, 6553-)**<br>N : 34 (0%, 10+, 24-)<br>---------------<br>DEL: 155<br>INS: 215	chr1:161.626.250<br>Total count: 10345<br>T: 83 (1%, 42+, 41-)<br>G: 21 (0%, 15+, 6-)<br>**C: 10179 (98%, 3717+, 6462-)**<br>A: 23 (0%, 21+, 2-)<br>N : 39 (0%, 12+, 27-)<br>---------------<br>DEL: 738<br>INS: 238	chr1:161.626.196<br>Total count: 10913<br>T: 10 (0%, 1+, 9-)<br>**G: 10742 (98%, 3987+, 6755-)**<br>C: 27 (0%, 16+, 11-)<br>A: 99 (1%, 32+, 67-)<br>N : 35 (0%, 12+, 23-)<br>---------------<br>DEL: 170<br>INS: 25	chr1:161.624.609<br>Total count: 11039<br>T: 37 (0%, 32+, 5-)<br>G: 2 (0%, 0+, 2-)<br>**C: 10930 (99%, 2100+, 8830-)**<br>A: 16 (0%, 0+, 16-)<br>N : 54 (0%, 6+, 48-)<br>---------------<br>DEL: 196<br>INS: 43	chr1:161.624.517<br>Total count: 10969<br>**T: 10822 (99%, 2080+, 8742-)**<br>G: 21 (0%, 3+, 18-)<br>C: 55 (1%, 7+, 48-)<br>A: 23 (0%, 6+, 17-)<br>N : 48 (0%, 5+, 43-)<br>---------------<br>DEL: 259<br>INS: 564	**02/GCGCAA*
AAL_014	chr1:161.629.989<br>Total count: 70143<br>T: 1095 (2%, 949+, 146-)<br>**G: 39315 (56%, 32961+, 6354-)**<br>**C: 25489 (36%, 19822+, 5667-)**	chr1:161.629.983<br>Total count: 73485<br>**T: 32314 (44%, 26824+, 5490-)**<br>G: 1019 (1%, 874+, 145-)<br>**C: 35915 (49%, 27911+, 8004-)**	chr1:161.629.903<br>Total count: 75124<br>T: 320 (0%, 306+, 14-)<br>**G: 24539 (33%, 19670+, 4869-)**<br>C: 194 (0%, 146+, 48-)<br>**A: 46157**	chr1:161.629.864<br>Total count: 75371<br>T: 595 (1%, 443+, 152-)<br>G: 274 (0%, 144+, 130-)<br>**C: 45304 (60%, 36202+, 9102-)**<br>**A: 25242**	chr1:161.629.853<br>Total count: 75815<br>T: 649 (1%, 513+, 136-)<br>**G: 41492 (55%, 33215+, 8277-)**<br>C: 336 (0%, 269+, 67-)<br>**A: 29362**	chr1:161.629.781<br>Total count: 76038<br>T: 331 (0%, 320+, 11-)<br>**G: 21648 (28%, 17126+, 4522-)**<br>C: 318 (0%, 216+, 102-)<br>**A: 49583**	chr1:161.626.282<br>Total count: 76541<br>T: 154 (0%, 39+, 115-)<br>G: 1468 (2%, 118+, 1350-)<br>C: 203 (0%, 17+, 186-)<br>**A: 74424 (97%, 28718+,**	chr1:161.626.250<br>Total count: 72210<br>T: 541 (1%, 276+, 265-)<br>G: 144 (0%, 106+, 38-)<br>**C: 71052 (98%, 26244+, 44808-)**<br>A: 195 (0%,	chr1:161.626.196<br>Total count: 75860<br>T: 94 (0%, 26+, 68-)<br>**G: 74612 (98%, 27637+, 46975-)**<br>C: 179 (0%, 111+, 68-)<br>A: 701 (1%,	chr1:161.624.609<br>Total count: 69342<br>T: 376 (1%, 312+, 64-)<br>G: 34 (0%, 3+, 31-)<br>**C: 68379 (99%, 14720+, 53659-)**<br>A: 124 (0%,	chr1:161.624.517<br>Total count: 68612<br>**T: 67744 (99%, 14823+, 52921-)**<br>G: 89 (0%, 22+, 67-)<br>C: 301 (0%, 56+, 245-)<br>A: 94 (0%,	**01/*03/*04*

	A: 891 (1%, 478+, 413-)<br>N : 3353 (5%, 1708+, 1645-)<br>---------------<br>DEL: 6589<br>INS: 1652	A: 560 (1%, 321+, 239-)<br>N : 3677 (5%, 1778+, 1899-)<br>---------------<br>DEL: 3036<br>INS: 1746	**(61%, 36891+, 9266-)**<br>N : 3914 (5%, 1950+, 1964-)<br>---------------<br>DEL: 1913<br>INS: 1316	**(33%, 19658+, 5584-)**<br>N : 3956 (5%, 1957+, 1999-)<br>---------------<br>DEL: 1642<br>INS: 608	**(39%, 22110+, 7252-)**<br>N : 3976 (5%, 1951+, 2025-)<br>---------------<br>DEL: 1065<br>INS: 1800	**(65%, 37268+, 12315-)**<br>N : 4158 (5%, 2023+, 2135-)<br>---------------<br>DEL: 1606<br>INS: 897	**45706-)**<br>N : 292 (0%, 174+, 118-)<br>---------------<br>DEL: 1031<br>INS: 1454	161+, 34-)<br>N : 278 (0%, 159+, 119-)<br>---------------<br>DEL: 4958<br>INS: 1574	179+, 522-)<br>N : 274 (0%, 166+, 108-)<br>---------------<br>DEL: 1258<br>INS: 275	3+, 121-)<br>N : 429 (1%, 53+, 376-)<br>---------------<br>DEL: 1376<br>INS: 309	42+, 52-)<br>N : 384 (1%, 56+, 328-)<br>---------------<br>DEL: 1542<br>INS: 3567	
AAL_015	chr1:161.629.989<br>Total count: 74408<br>T: 1331 (2%, 1172+, 159-)<br>**G: 32633 (44%, 27458+, 5175-)**<br>**C: 37107 (50%, 29408+, 7699-)**<br>A: 924 (1%, 552+, 372-)<br>N : 2413 (3%, 1198+, 1215-)<br>---------------<br>DEL: 6254<br>INS: 1726	chr1:161.629.983<br>Total count: 77108<br>**T: 42852 (56%, 35458+, 7394-)**<br>G: 1152 (1%, 967+, 185-)<br>**C: 29923 (39%, 23151+, 6772-)**<br>A: 535 (1%, 272+, 263-)<br>N : 2646 (3%, 1273+, 1373-)<br>---------------<br>DEL: 3271<br>INS: 1619	chr1:161.629.903<br>Total count: 78505<br>T: 390 (0%, 379+, 11-)<br>**G: 36083 (46%, 29447+, 6636-)**<br>C: 185 (0%, 129+, 56-)<br>**A: 39033 (50%, 30839+, 8194-)**<br>N : 2814 (4%, 1402+, 1412-)<br>---------------<br>DEL: 2321<br>INS: 1385	chr1:161.629.864<br>Total count: 78956<br>T: 783 (1%, 627+, 156-)<br>G: 196 (0%, 104+, 92-)<br>**C: 56420 (71%, 44625+, 11795-)**<br>**A: 18741 (24%, 14965+, 3776-)**<br>N : 2816 (4%, 1397+, 1419-)<br>---------------<br>DEL: 1818<br>INS: 514	chr1:161.629.853<br>Total count: 79663<br>T: 514 (1%, 369+, 145-)<br>**G: 34496 (43%, 27425+, 7071-)**<br>C: 351 (0%, 286+, 65-)<br>**A: 41437 (52%, 31954+, 9483-)**<br>N : 2865 (4%, 1402+, 1463-)<br>---------------<br>DEL: 875<br>INS: 1617	chr1:161.629.781<br>Total count: 79526<br>T: 331 (0%, 324+, 7-)<br>**G: 18979 (24%, 14971+, 4008-)**<br>C: 337 (0%, 230+, 107-)<br>**A: 56923 (72%, 43062+, 13861-)**<br>N : 2956 (4%, 1435+, 1521-)<br>---------------<br>DEL: 1771<br>INS: 891	chr1:161.626.282<br>Total count: 80965<br>T: 137 (0%, 39+, 98-)<br>G: 1487 (2%, 100+, 1387-)<br>C: 227 (0%, 17+, 210-)<br>A: 78833 (97%, 28341+, 50492-)<br>N : 281 (0%, 151+, 130-)<br>---------------<br>DEL: 1116<br>INS: 1489	chr1:161.626.250<br>Total count: 76496<br>T: 610 (1%, 295+, 315-)<br>G: 160 (0%, 112+, 48-)<br>C: 75281 (98%, 25964+, 49317-)<br>A: 176 (0%, 123+, 53-)<br>N : 269 (0%, 149+, 120-)<br>---------------<br>DEL: 5303<br>INS: 1655	chr1:161.626.196<br>Total count: 80467<br>T: 113 (0%, 32+, 81-)<br>G: 79169 (98%, 27347+, 51822-)<br>C: 184 (0%, 113+, 71-)<br>A: 728 (1%, 192+, 536-)<br>N : 273 (0%, 155+, 118-)<br>---------------<br>DEL: 1314<br>INS: 275	chr1:161.624.609<br>Total count: 78686<br>T: 306 (0%, 259+, 47-)<br>G: 45 (0%, 5+, 40-)<br>C: 77827 (99%, 15024+, 62803-)<br>A: 128 (0%, 3+, 125-)<br>N : 380 (0%, 46+, 334-)<br>---------------<br>DEL: 1380<br>INS: 339	chr1:161.624.517<br>Total count: 78251<br>T: 77417 (99%, 15057+, 62360-)<br>G: 133 (0%, 31+, 102-)<br>C: 266 (0%, 38+, 228-)<br>A: 90 (0%, 38+, 52-)<br>N : 345 (0%, 42+, 303-)<br>---------------<br>DEL: 1735<br>INS: 3999	**01/*02/*03/*04*
AAL_016	chr1:161.629.989<br>Total count: 11530<br>T: 0<br>G: 28 (0%, 24+, 4-)<br>C: 49 (0%, 37+, 12-)<br>A: 1 (0%, 0+, 1-)<br>**N : 11452 (99%, 9598+, 1854-)**<br>---------------<br>DEL: 1358<br>INS: 267	chr1:161.629.983<br>Total count: 12428<br>T: 54 (0%, 43+, 11-)<br>G: 4 (0%, 4+, 0-)<br>C: 24 (0%, 18+, 6-)<br>A: 0<br>**N : 12346 (99%, 10274+, 2072-)**<br>---------------<br>DEL: 466<br>INS: 316	chr1:161.629.903<br>Total count: 12242<br>T: 1 (0%, 1+, 0-)<br>G: 43 (0%, 34+, 9-)<br>C: 0<br>A: 38 (0%, 29+, 9-)<br>**N : 12160 (99%, 10233+, 1927-)**<br>---------------<br>DEL: 544<br>INS: 208	chr1:161.629.864<br>Total count: 12518<br>T: 0<br>G: 1 (0%, 1+, 0-)<br>C: 72 (1%, 56+, 16-)<br>A: 10 (0%, 7+, 3-)<br>**N : 12435 (99%, 10243+, 2192-)**<br>---------------<br>DEL: 251<br>INS: 36	chr1:161.629.853<br>Total count: 12537<br>T: 1 (0%, 1+, 0-)<br>G: 38 (0%, 28+, 10-)<br>C: 0<br>A: 44 (0%, 34+, 10-)<br>**N : 12454 (99%, 10249+, 2205-)**<br>---------------<br>DEL: 205<br>INS: 477	chr1:161.629.781<br>Total count: 12528<br>T: 0<br>G: 21 (0%, 17+, 4-)<br>C: 0<br>A: 62 (0%, 44+, 18-)<br>**N : 12445 (99%, 9968+, 2477-)**<br>---------------<br>DEL: 352<br>INS: 87	chr1:161.626.282<br>Total count: 12589<br>T: 0<br>G: 1 (0%, 0+, 1-)<br>C: 0<br>A: 95 (1%, 31+, 64-)<br>**N : 12493 (99%, 4755+, 7738-)**<br>---------------<br>DEL: 845<br>INS: 342	chr1:161.626.250<br>Total count: 12131<br>T: 0<br>G: 0<br>C: 91 (1%, 30+, 61-)<br>A: 1 (0%, 0+, 1-)<br>**N : 12039 (99%, 4206+, 7833-)**<br>---------------<br>DEL: 1322<br>INS: 431	chr1:161.626.196<br>Total count: 10499<br>T: 1 (0%, 0+, 1-)<br>G: 94 (1%, 31+, 63-)<br>C: 0<br>A: 0<br>**N : 10404 (99%, 4098+, 6306-)**<br>---------------<br>DEL: 3014<br>INS: 28	chr1:161.624.609<br>Total count: 12652<br>T: 3 (0%, 1+, 2-)<br>G: 0<br>C: 94 (1%, 18+, 76-)<br>A: 0<br>**N : 12555 (99%, 2355+, 10200-)**<br>---------------<br>DEL: 291<br>INS: 19	chr1:161.624.517<br>Total count: 12517<br>T: 90 (1%, 16+, 74-)<br>G: 0<br>C: 2 (0%, 0+, 2-)<br>A: 0<br>**N : 12425 (99%, 2287+, 10138-)**<br>---------------<br>DEL: 288<br>INS: 1168	Null/Null

Table S4 Long-range sequencing raw data for 11 SNPs in 10 samples from NHSBT

**New**	**c.108**	**c.114**	**c.194**	**c.233**	**c.244**	**c.316**					**STOP CODON**	
**Old**	**c.141**	**c.147**	**c.227**	**c.266**	**c.277**	**c.349**	**c.473**	**c.505**	**c.559**	**c.641**	**c.733**	
Sample ID	rs200688856	rs527909462	rs448740	rs5030738	rs147574249	rs2290834	rs71632959	rs71632958	rs200215055	rs758550229	rs374752953	*FCGR3B* alleles
BRI_001	chr1:161.629.989	chr1:161.629.983	chr1:161.629.903	chr1:161.629.864	chr1:161.629.853	chr1:161.629.781	chr1:161.626.282	chr1:161.626.250	chr1:161.626.196	chr1:161.624.609	chr1:161.624.517	**01/*02*
	Total count: 82644<br>T : 1538 (2%, 1317+, 221-)<br>**G : 34787 (42%, 29106+, 5681-)**<br>**C : 42090 (51%, 33330+, 8760-)**<br>A : 979 (1%, 587+, 392-)<br>N : 3250 (4%, 1937+, 1313-)<br>---------------<br>DEL: 6934<br>INS: 1798	Total count: 85829<br>**T : 48983 (57%, 40766+, 8217-)**<br>G : 1217 (1%, 942+, 275-)<br>**C : 31347 (37%, 24129+, 7218-)**<br>A : 793 (1%, 352+, 441-)<br>N : 3489 (4%, 2022+, 1467-)<br>---------------<br>DEL: 3569<br>INS: 1625	Total count: 86930<br>T : 373 (0%, 361+, 12-)<br>**G : 41466 (48%, 33856+, 7610-)**<br>C : 252 (0%, 182+, 70-)<br>**A : 41262 (47%, 32699+, 8563-)**<br>N : 3577 (4%, 2126+, 1451-)<br>---------------<br>DEL: 2877<br>INS: 1659	Total count: 87805<br>T : 983 (1%, 816+, 167-)<br>G : 55 (0%, 50+, 5-)<br>**C : 82618 (94%, 65321+, 17297-)**<br>A : 482 (1%, 451+, 31-)<br>N : 3667 (4%, 2161+, 1506-)<br>---------------<br>DEL: 2048<br>INS: 402	Total count: 88622<br>T : 639 (1%, 477+, 162-)<br>**G : 36903 (42%, 29416+, 7487-)**<br>C : 413 (0%, 357+, 56-)<br>**A : 46988 (53%, 36423+, 10565-)**<br>N : 3679 (4%, 2131+, 1548-)<br>---------------<br>DEL: 1047<br>INS: 1792	Total count: 89717<br>T : 401 (0%, 396+, 5-)<br>**G : 38841 (43%, 30293+, 8548-)**<br>C : 375 (0%, 251+, 124-)<br>**A : 46297 (52%, 34616+, 11681-)**<br>N : 3803 (4%, 2131+, 1672-)<br>---------------<br>DEL: 1722<br>INS: 1330	Total count: 91576<br>T : 166 (0%, 46+, 120-)<br>G : 1794 (2%, 166+, 1628-)<br>C : 240 (0%, 16+, 224-)<br>**A : 87602 (96%, 32629+, 54973-)**<br>N : 1774 (2%, 789+, 985-)<br>---------------<br>DEL: 1432<br>INS: 2032	Total count: 86469<br>T : 866 (1%, 490+, 376-)<br>G : 234 (0%, 166+, 68-)<br>**C : 83446 (97%, 29411+, 54035-)**<br>A : 200 (0%, 153+, 47-)<br>N : 1723 (2%, 712+, 1011-)<br>---------------<br>DEL: 6486<br>INS: 1667	Total count: 90596<br>T : 200 (0%, 73+, 127-)<br>**G : 87847 (97%, 31992+, 55855-)**<br>C : 256 (0%, 164+, 92-)<br>A : 967 (1%, 240+, 727-)<br>N : 1326 (1%, 632+, 694-)<br>---------------<br>DEL: 2318<br>INS: 293	Total count: 90861<br>T : 857 (1%, 592+, 265-)<br>G : 64 (0%, 10+, 54-)<br>**C : 87566 (96%, 18661+, 68905-)**<br>A : 169 (0%, 5+, 164-)<br>N : 2205 (2%, 403+, 1802-)<br>---------------<br>DEL: 2085<br>INS: 375	Total count: 90593<br>**T : 87532 (97%, 19239+, 68293-)**<br>G : 171 (0%, 30+, 141-)<br>C : 663 (1%, 214+, 449-)<br>A : 164 (0%, 98+, 66-)<br>N : 2063 (2%, 409+, 1654-)<br>---------------<br>DEL: 2148<br>INS: 4735	
BRI_002	chr1:161.629.989	chr1:161.629.983	chr1:161.629.903	chr1:161.629.864	chr1:161.629.853	chr1:161.629.781	chr1:161.626.282	chr1:161.626.250	chr1:161.626.196	chr1:161.624.609	chr1:161.624.517	**02/*03/*04*
	Total count: 41837<br>T: 823 (2%, 693+, 130-)<br>**G: 12298 (29%, 10062+, 2236-)**<br>**C: 26721 (64%, 20722+, 5999-)**<br>A: 467 (1%, 318+, 149-)<br>N : 1528 (4%, 767+, 761-)<br>---------------<br>DEL: 2942<br>INS: 864	Total count: 42850<br>**T: 28882 (67%, 23290+, 5592-)**<br>G: 697 (2%, 511+, 186-)<br>**C: 11196 (26%, 8311+, 2885-)**<br>A: 433 (1%, 135+, 298-)<br>N : 1642 (4%, 798+, 844-)<br>---------------<br>DEL: 1850<br>INS: 750	Total count: 43597<br>T: 187 (0%, 182+, 5-)<br>**G: 26328 (60%, 21167+, 5161-)**<br>C: 123 (0%, 93+, 30-)<br>**A: 15161 (35%, 11280+, 3881-)**<br>N : 1798 (4%, 910+, 888-)<br>---------------<br>DEL: 1646<br>INS: 914	Total count: 44124<br>T: 361 (1%, 286+, 75-)<br>G: 157 (0%, 72+, 85-)<br>**C: 28348 (64%, 21684+, 6664-)**<br>**A: 13435 (30%, 10409+, 3026-)**<br>N : 1823 (4%, 918+, 905-)<br>---------------<br>DEL: 1112<br>INS: 339	Total count: 44705<br>T: 252 (1%, 172+, 80-)<br>**G: 13044 (29%, 9923+, 3121-)**<br>**C: 223 (0%, 190+, 33-)**<br>**A: 29344 (66%, 22210+,** 7134-)<br>N : 1842 (4%, 917+, 925-)<br>---------------<br>DEL: 417<br>INS: 644	Total count: 44519<br>T: 241 (1%, 238+, 3-)<br>G: 429 (1%, 296+, 133-)<br>C: 217 (0%, 145+, 72-)<br>**A: 41738 (94%, 30966+, 10772-)**<br>N : 1894 (4%, 934+, 960-)<br>---------------<br>DEL: 1167<br>INS: 301	Total count: 45754<br>T: 86 (0%, 38+, 48-)<br>G: 844 (2%, 88+, 756-)<br>C: 108 (0%, 8+, 100-)<br>**A: 44298 (97%, 17606+, 26692-)**<br>N : 418 (1%, 223+, 195-)<br>---------------<br>DEL: 679<br>INS: 934	Total count: 43046<br>T: 413 (1%, 255+, 158-)<br>G: 97 (0%, 74+, 23-)<br>**C: 42044 (98%, 15882+, 26162-)**<br>A: 106 (0%, 83+, 23-)<br>N : 386 (1%, 191+, 195-)<br>---------------<br>DEL: 3186<br>INS: 812	Total count: 45476<br>T: 69 (0%, 16+, 53-)<br>**G: 44421 (98%, 16990+, 27431-)**<br>C: 120 (0%, 73+, 47-)<br>A: 486 (1%, 121+, 365-)<br>N : 380 (1%, 202+, 178-)<br>---------------<br>DEL: 843<br>INS: 136	Total count: 42959<br>T: 271 (1%, 209+, 62-)<br>G: 34 (0%, 5+, 29-)<br>**C: 42115 (98%, 9538+, 32577-)**<br>A: 79 (0%, 3+, 76-)<br>N : 460 (1%, 86+, 374-)<br>---------------<br>DEL: 1041<br>INS: 184	Total count: 42893<br>**T: 42146 (98%, 9767+, 32379-)**<br>G: 69 (0%, 19+, 50-)<br>C: 190 (0%, 45+, 145-)<br>A: 62 (0%, 34+, 28-)<br>N : 426 (1%, 88+, 338-)<br>---------------<br>DEL: 895<br>INS: 2264	
BRI_003	chr1:161.629.989	chr1:161.629.983	chr1:161.629.903	chr1:161.629.864	chr1:161.629.853	chr1:161.629.781	chr1:161.626.282	chr1:161.626.250	chr1:161.626.196	chr1:161.624.609	chr1:161.624.517	**01/*03*
	Total count: 106509	Total count: 110569	Total count: 112680	Total count: 113629	Total count: 114760	Total count: 115870	Total count: 119779	Total count: 113005	Total count: 119271	Total count: 113491	Total count: 112969	

	T: 1824 (2%, 1561+, 263-)<br>**G: 45604 (43%, 37504+, 8100-)**<br>**C: 53720 (50%, 41255+, 12465-)**<br>A: 1248 (1%, 752+, 496-)<br>N : 4113 (4%, 1827+, 2286-)<br>---------------<br>DEL: 9007<br>INS: 2340	**T: 62092 (56%, 50467+, 11625-)**<br>G: 1574 (1%, 1172+, 402-)<br>**C: 41355 (37%, 31093+, 10262-)**<br>A: 1031 (1%, 430+, 601-)<br>N : 4517 (4%, 1932+, 2585-)<br>---------------<br>DEL: 4614<br>INS: 2098	T: 523 (0%, 509+, 14-)<br>**G: 52091 (46%, 41473+, 10618-)**<br>C: 281 (0%, 199+, 82-)<br>**A: 54984 (49%, 42390+, 12594-)**<br>N : 4801 (4%, 2123+, 2678-)<br>---------------<br>DEL: 3651<br>INS: 2121	T: 835 (1%, 606+, 229-)<br>G: 541 (0%, 238+, 303-)<br>**C: 53641 (47%, 41682+, 11959-)**<br>**A: 53779 (47%, 41487+, 12292-)**<br>N : 4833 (4%, 2126+, 2707-)<br>---------------<br>DEL: 2659<br>INS: 1059	T: 786 (1%, 604+, 182-)<br>**G: 48752 (42%, 37884+, 10868-)**<br>C: 511 (0%, 436+, 75-)<br>**A: 59828 (52%, 44798+, 15030-)**<br>N : 4883 (4%, 2122+, 2761-)<br>---------------<br>DEL: 1246<br>INS: 2148	T: 473 (0%, 456+, 17-)<br>**G: 51384 (44%, 38933+, 12451-)**<br>C: 451 (0%, 318+, 133-)<br>**A: 58451 (50%, 42535+, 15916-)**<br>N : 5111 (4%, 2218+, 2893-)<br>---------------<br>DEL: 2005<br>INS: 1618	T: 218 (0%, 69+, 149-)<br>G: 2108 (2%, 124+, 1984-)<br>C: 283 (0%, 24+, 259-)<br>**A: 116799 (98%, 44133+, 72666-)**<br>N : 371 (0%, 223+, 148-)<br>---------------<br>DEL: 1751<br>INS: 2630	T: 962 (1%, 593+, 369-)<br>G: 282 (0%, 207+, 75-)<br>**C: 111137 (98%, 39747+, 71390-)**<br>A: 279 (0%, 204+, 75-)<br>N : 345 (0%, 213+, 132-)<br>---------------<br>DEL: 8499<br>INS: 2284	T: 124 (0%, 42+, 82-)<br>**G: 117228 (98%, 43371+, 73857-)**<br>C: 351 (0%, 223+, 128-)<br>A: 1201 (1%, 260+, 941-)<br>N : 367 (0%, 223+, 144-)<br>---------------<br>DEL: 2134<br>INS: 390	T: 500 (0%, 451+, 49-)<br>G: 80 (0%, 8+, 72-)<br>**C: 111997 (99%, 25012+, 86985-)**<br>A: 218 (0%, 5+, 213-)<br>N : 696 (1%, 87+, 609-)<br>---------------<br>DEL: 2535<br>INS: 400	**T: 111704 (99%, 25456+, 86248-)**<br>G: 176 (0%, 38+, 138-)<br>C: 336 (0%, 70+, 266-)<br>A: 144 (0%, 72+, 72-)<br>N : 609 (1%, 95+, 514-)<br>---------------<br>DEL: 2506<br>INS: 5796	
	chr1:161.629.989	chr1:161.629.983	chr1:161.629.903	chr1:161.629.864	chr1:161.629.853	chr1:161.629.781	chr1:161.626.282	chr1:161.626.250	chr1:161.626.196	chr1:161.624.609	chr1:161.624.517	
BRI_004	Total count: 102351<br>T: 1757 (2%, 1506+, 251-)<br>**G: 42183 (41%, 34475+, 7708-)**<br>**C: 51485 (50%, 39610+, 11875-)**<br>A: 1214 (1%, 685+, 529-)<br>N : 5712 (6%, 3172+, 2540-)<br>---------------<br>DEL: 8621<br>INS: 2270	Total count: 106256<br>**T: 59158 (56%, 47952+, 11206-)**<br>G: 1510 (1%, 1133+, 377-)<br>**C: 38477 (36%, 28690+, 9787-)**<br>A: 934 (1%, 367+, 567-)<br>N : 6177 (6%, 3357+, 2820-)<br>---------------<br>DEL: 4500<br>INS: 2002	Total count: 108325<br>T: 462 (0%, 445+, 17-)<br>**G: 50474 (47%, 40227+, 10247-)**<br>C: 294 (0%, 202+, 92-)<br>**A: 50677 (47%, 38759+, 11918-)**<br>N : 6418 (6%, 3550+, 2868-)<br>---------------<br>DEL: 3633<br>INS: 2103	Total count: 109417<br>T: 1145 (1%, 940+, 205-)<br>G: 69 (0%, 55+, 14-)<br>**C: 101141 (92%, 77572+, 23569-)**<br>A: 536 (0%, 512+, 24-)<br>N : 6526 (6%, 3590+, 2936-)<br>---------------<br>DEL: 2573<br>INS: 489	Total count: 110554<br>T: 691 (1%, 505+, 186-)<br>**G: 45287 (41%, 34941+, 10346-)**<br>C: 482 (0%, 388+, 94-)<br>**A: 57501 (52%, 43163+, 14338-)**<br>N : 6593 (6%, 3587+, 3006-)<br>---------------<br>DEL: 1173<br>INS: 2119	Total count: 111662<br>T: 486 (0%, 468+, 18-)<br>**G: 47057 (42%, 35676+, 11381-)**<br>C: 475 (0%, 299+, 176-)<br>**A: 56875 (51%, 41169+, 15706-)**<br>N : 6769 (6%, 3607+, 3162-)<br>---------------<br>DEL: 2063<br>INS: 1534	Total count: 116932<br>T: 221 (0%, 64+, 157-)<br>G: 2424 (2%, 254+, 2170-)<br>C: 282 (0%, 17+, 265-)<br>**A: 111874 (96%, 43394+, 68480-)**<br>N : 2131 (2%, 951+, 1180-)<br>---------------<br>DEL: 1774<br>INS: 2523	Total count: 110305<br>T: 1172 (1%, 645+, 527-)<br>G: 267 (0%, 193+, 74-)<br>**C: 106527 (97%, 39109+, 67418-)**<br>A: 307 (0%, 234+, 73-)<br>N : 2032 (2%, 836+, 1196-)<br>---------------<br>DEL: 8281<br>INS: 2237	Total count: 116170<br>T: 208 (0%, 73+, 135-)<br>**G: 112450 (97%, 42725+, 69725-)**<br>C: 290 (0%, 168+, 122-)<br>A: 1308 (1%, 328+, 980-)<br>N : 1914 (2%, 856+, 1058-)<br>---------------<br>DEL: 2396<br>INS: 395	Total count: 109307<br>T: 961 (1%, 639+, 322-)<br>G: 68 (0%, 3+, 65-)<br>**C: 105498 (97%, 22836+, 82662-)**<br>A: 233 (0%, 5+, 228-)<br>N : 2547 (2%, 510+, 2037-)<br>---------------<br>DEL: 2444<br>INS: 462	Total count: 108668<br>**T: 105149 (97%, 23353+, 81796-)**<br>G: 186 (0%, 48+, 138-)<br>C: 771 (1%, 232+, 539-)<br>A: 165 (0%, 95+, 70-)<br>N : 2397 (2%, 516+, 1881-)<br>---------------<br>DEL: 2512<br>INS: 5853	*01/*02
	chr1:161.629.989	chr1:161.629.983	chr1:161.629.903	chr1:161.629.864	chr1:161.629.853	chr1:161.629.781	chr1:161.626.282	chr1:161.626.250	chr1:161.626.196	chr1:161.624.609	chr1:161.624.517	
BRI_005	Total count: 102351<br>T: 1757 (2%, 1506+, 251-)<br>**G: 42183 (41%, 34475+, 7708-)**<br>**C: 51485 (50%, 39610+, 11875-)**<br>A: 1214 (1%, 685+, 529-)<br>N : 5712 (6%, 3172+, 2540-)<br>---------------<br>DEL: 8621<br>INS: 2270	Total count: 106256<br>**T: 59158 (56%, 47952+, 11206-)**<br>G: 1510 (1%, 1133+, 377-)<br>**C: 38477 (36%, 28690+, 9787-)**<br>A: 934 (1%, 367+, 567-)<br>N : 6177 (6%, 3357+, 2820-)<br>---------------<br>DEL: 4500<br>INS: 2002	Total count: 108325<br>T: 462 (0%, 445+, 17-)<br>**G: 50474 (47%, 40227+, 10247-)**<br>C: 294 (0%, 202+, 92-)<br>**A: 50677 (47%, 38759+, 11918-)**<br>N : 6418 (6%, 3550+, 2868-)<br>---------------<br>DEL: 3633<br>INS: 2103	Total count: 109417<br>T: 1145 (1%, 940+, 205-)<br>G: 69 (0%, 55+, 14-)<br>**C: 101141 (92%, 77572+, 23569-)**<br>A: 536 (0%, 512+, 24-)<br>N : 6526 (6%, 3590+, 2936-)<br>---------------<br>DEL: 2573<br>INS: 489	Total count: 110554<br>T: 691 (1%, 505+, 186-)<br>**G: 45287 (41%, 34941+, 10346-)**<br>C: 482 (0%, 388+, 94-)<br>**A: 57501 (52%, 43163+, 14338-)**<br>N : 6593 (6%, 3587+, 3006-)<br>---------------<br>DEL: 1173<br>INS: 2119	Total count: 111662<br>T: 486 (0%, 468+, 18-)<br>**G: 47057 (42%, 35676+, 11381-)**<br>C: 475 (0%, 299+, 176-)<br>**A: 56875 (51%, 41169+, 15706-)**<br>N : 6769 (6%, 3607+, 3162-)<br>---------------<br>DEL: 2063<br>INS: 1534	Total count: 116932<br>T: 221 (0%, 64+, 157-)<br>G: 2424 (2%, 254+, 2170-)<br>C: 282 (0%, 17+, 265-)<br>**A: 111874 (96%, 43394+, 68480-)**<br>N : 2131 (2%, 951+, 1180-)<br>---------------<br>DEL: 1774<br>INS: 2523	Total count: 110305<br>T: 1172 (1%, 645+, 527-)<br>G: 267 (0%, 193+, 74-)<br>**C: 106527 (97%, 39109+, 67418-)**<br>A: 307 (0%, 234+, 73-)<br>N : 2032 (2%, 836+, 1196-)<br>---------------<br>DEL: 8281<br>INS: 2237	Total count: 116170<br>T: 208 (0%, 73+, 135-)<br>**G: 112450 (97%, 42725+, 69725-)**<br>C: 290 (0%, 168+, 122-)<br>A: 1308 (1%, 328+, 980-)<br>N : 1914 (2%, 856+, 1058-)<br>---------------<br>DEL: 2396<br>INS: 395	Total count: 109307<br>T: 961 (1%, 639+, 322-)<br>G: 68 (0%, 3+, 65-)<br>**C: 105498 (97%, 22836+, 82662-)**<br>A: 233 (0%, 5+, 228-)<br>N : 2547 (2%, 510+, 2037-)<br>---------------<br>DEL: 2444<br>INS: 462	Total count: 108668<br>**T: 105149 (97%, 23353+, 81796-)**<br>G: 186 (0%, 48+, 138-)<br>C: 771 (1%, 232+, 539-)<br>A: 165 (0%, 95+, 70-)<br>N : 2397 (2%, 516+, 1881-)<br>---------------<br>DEL: 2512<br>INS: 5853	*01/*02

	chr1:161.629.<br>989	chr1:161.629.<br>983	chr1:161.629.<br>903	chr1:161.629.<br>864	chr1:161.629.<br>853	chr1:161.629.<br>781	chr1:161.626.<br>282	chr1:161.626.<br>250	chr1:161.626.<br>196	chr1:161.624.<br>609	chr1:161.624.<br>517	
BRI_0<br>06	Total count:<br>1931<br>T: 0<br>G: 38 (2%,<br>29+, 9-)<br>C: 31 (2%,<br>25+, 6-)<br>A: 2 (0%, 1+,<br>1-)<br>**N : 1860 (96%,<br>1607+, 253-)**<br>---------------<br>DEL: 241<br>INS: 26	Total count:<br>2105<br>T: 35 (2%, 29+,<br>6-)<br>G: 2 (0%, 1+,<br>1-)<br>C: 33 (2%,<br>23+, 10-)<br>A: 1 (0%, 1+,<br>0-)<br>**N : 2034 (97%,<br>1737+, 297-)**<br>---------------<br>DEL: 70<br>INS: 43	Total count:<br>2089<br>T: 1 (0%, 1+, 0-<br>)<br>G: 32 (2%,<br>28+, 4-)<br>C: 0<br>A: 35 (2%,<br>24+, 11-)<br>**N : 2021 (97%,<br>1739+, 282-)**<br>---------------<br>DEL: 87<br>INS: 49	Total count:<br>2134<br>T: 2 (0%, 1+, 1-<br>)<br>G: 0<br>C: 54 (3%,<br>40+, 14-)<br>A: 13 (1%,<br>11+, 2-)<br>**N : 2065 (97%,<br>1732+, 333-)**<br>---------------<br>DEL: 46<br>INS: 7	Total count:<br>2152<br>T: 0<br>G: 35 (2%,<br>25+, 10-)<br>C: 1 (0%, 1+,<br>0-)<br>A: 34 (2%,<br>26+, 8-)<br>**N : 2082 (97%,<br>1738+, 344-)**<br>---------------<br>DEL: 29<br>INS: 75	Total count:<br>2088<br>T: 0<br>G: 22 (1%,<br>16+, 6-)<br>C: 0<br>A: 46 (2%,<br>34+, 12-)<br>**N : 2020 (97%,<br>1652+, 368-)**<br>---------------<br>DEL: 56<br>INS: 23	Total count:<br>2015<br>T: 0<br>G: 1 (0%, 1+,<br>0-)<br>C: 0<br>A: 75 (4%,<br>24+, 51-)<br>**N : 1939 (96%,<br>714+, 1225-)**<br>---------------<br>DEL: 134<br>INS: 54	Total count:<br>1900<br>T: 0<br>G: 0<br>C: 74 (4%,<br>25+, 49-)<br>A: 0<br>**N : 1826 (96%,<br>599+, 1227-)**<br>---------------<br>DEL: 250<br>INS: 55	Total count:<br>2124<br>T: 0<br>G: 76 (4%,<br>25+, 51-)<br>C: 0<br>A: 1 (0%, 0+,<br>1-)<br>**N : 2047 (96%,<br>741+, 1306-)**<br>---------------<br>DEL: 35<br>INS: 8	Total count:<br>2081<br>T: 0<br>G: 0<br>C: 83 (4%,<br>17+, 66-)<br>A: 0<br>**N : 1998 (96%,<br>366+, 1632-)**<br>---------------<br>DEL: 49<br>INS: 1	Total count:<br>2056<br>T: 80 (4%, 14+,<br>66-)<br>G: 0<br>C: 0<br>A: 0<br>**N : 1976 (96%,<br>351+, 1625-)**<br>---------------<br>DEL: 49<br>INS: 193	Null/Null
	chr1:161.629.<br>989	chr1:161.629.<br>983	chr1:161.629.<br>903	chr1:161.629.<br>864	chr1:161.629.<br>853	chr1:161.629.<br>781	chr1:161.626.<br>282	chr1:161.626.<br>250	chr1:161.626.<br>196	chr1:161.624.<br>609	chr1:161.624.<br>517	GCGCGAA+<br>rs761007181<br>chr1:161.629.<br>900
BRI_0<br>07	Total count:<br>51786<br>T: 388 (1%,<br>356+, 32-)<br>**G: 43123<br>(83%, 35536+,<br>7587-)**<br>C: 580 (1%,<br>373+, 207-)<br>A: 757 (1%,<br>331+, 426-)<br>N : 6938 (13%,<br>4848+, 2090-)<br>---------------<br>DEL: 6545<br>INS: 1230	Total count:<br>56295<br>T: 8641 (15%,<br>8364+, 277-)<br>G: 470 (1%,<br>457+, 13-)<br>**C: 39366<br>(70%, 30382+,<br>8984-)**<br>A: 262 (0%,<br>221+, 41-)<br>N : 7556 (13%,<br>5181+, 2375-)<br>---------------<br>DEL: 2041<br>INS: 1371	Total count:<br>55226<br>T: 168 (0%,<br>163+, 5-)<br>**G: 45940<br>(83%, 38331+,<br>7609-)**<br>C: 80 (0%,<br>38+, 42-)<br>A: 1694 (3%,<br>903+, 791-)<br>N : 7344 (13%,<br>5242+, 2102-)<br>---------------<br>DEL: 3089<br>INS: 878	Total count:<br>57032<br>T: 662 (1%,<br>593+, 69-)<br>G: 41 (0%,<br>33+, 8-)<br>**C: 48439<br>(85%, 38759+,<br>9680-)**<br>A: 259 (0%,<br>246+, 13-)<br>N : 7631 (13%,<br>5263+, 2368-)<br>---------------<br>DEL: 1364<br>INS: 232	Total count:<br>57324<br>T: 532 (1%,<br>439+, 93-)<br>**G: 45739<br>(80%, 36778+,<br>8961-)**<br>C: 236 (0%,<br>195+, 41-)<br>A: 3187 (6%,<br>2452+, 735-)<br>N : 7630 (13%,<br>5282+, 2348-)<br>---------------<br>DEL: 970<br>INS: 2245	Total count:<br>57599<br>T: 297 (1%,<br>293+, 4-)<br>G: 513 (1%,<br>367+, 146-)<br>C: 308 (1%,<br>216+, 92-)<br>**A: 48874<br>(85%, 37997+,<br>10877-)**<br>N : 7607 (13%,<br>5162+, 2445-)<br>---------------<br>DEL: 1649<br>INS: 453	Total count:<br>60105<br>T: 101 (0%,<br>37+, 64-)<br>G: 1480 (2%,<br>325+, 1155-)<br>C: 138 (0%,<br>16+, 122-)<br>**A: 53314<br>(89%, 22066+,**<br>31248-)<br>N : 5072 (8%,<br>1962+, 3110-)<br>---------------<br>DEL: 1188<br>INS: 1317	Total count:<br>56871<br>T: 910 (2%,<br>464+, 446-)<br>G: 137 (0%,<br>111+, 26-)<br>**C: 50811<br>(89%, 19946+,<br>30865-)**<br>A: 150 (0%,<br>111+, 39-)<br>N : 4863 (9%,<br>1728+, 3135-)<br>---------------<br>DEL: 4476<br>INS: 1182	Total count:<br>59233<br>T: 200 (0%,<br>73+, 127-)<br>**G: 53874<br>(91%, 21749+,<br>32125-)**<br>C: 169 (0%,<br>128+, 41-)<br>A: 587 (1%,<br>154+, 433-)<br>N : 4403 (7%,<br>1749+, 2654-)<br>---------------<br>DEL: 2161<br>INS: 212	Total count:<br>60207<br>T: 1267 (2%,<br>665+, 602-)<br>G: 42 (0%, 1+,<br>41-)<br>**C: 53297<br>(89%, 12036+,<br>41261-)**<br>A: 112 (0%,<br>3+, 109-)<br>N : 5489 (9%,<br>1244+, 4245-)<br>---------------<br>DEL: 1399<br>INS: 245	Total count:<br>60090<br>**T: 53422<br>(89%, 12322+,**<br>41100-)<br>G: 100 (0%,<br>17+, 83-)<br>C: 1092 (2%,<br>436+, 656-)<br>A: 102 (0%,<br>52+, 50-)<br>N : 5374 (9%,<br>1274+, 4100-)<br>---------------<br>DEL: 1405<br>INS: 3220	Total count:<br>55895<br>T: 3213 (6%,<br>2766+, 447-)<br>**G: 43878<br>(79%, 35831+,<br>8047-)**<br>C: 174 (0%,<br>140+, 34-)<br>A: 1115 (2%,<br>526+, 589-)<br>N : 7515 (13%,<br>5253+, 2262-)<br>---------------<br>DEL: 2444<br>INS: 1508
	chr1:161.629.<br>989	chr1:161.629.<br>983	chr1:161.629.<br>903	chr1:161.629.<br>864	chr1:161.629.<br>853	chr1:161.629.<br>781	chr1:161.626.<br>282	chr1:161.626.<br>250	chr1:161.626.<br>196	chr1:161.624.<br>609	chr1:161.624.<br>517	
BRI_0<br>08	Total count:<br>74368<br>T: 600 (1%,<br>569+, 31-)<br>**G: 63397<br>(85%, 52377+,<br>11020-)**<br>C: 784 (1%,	Total count:<br>80510<br>T: 13214<br>(16%, 12855+,<br>359-)<br>G: 839 (1%,<br>818+, 21-)<br>**C: 56880**	Total count:<br>82570<br>T: 291 (0%,<br>287+, 4-)<br>G: 1521 (2%,<br>933+, 588-)<br>C: 220 (0%,<br>179+, 41-)	Total count:<br>81976<br>T: 594 (1%,<br>496+, 98-)<br>G: 39 (0%,<br>33+, 6-)<br>**C: 71565<br>(87%, 57524+,**	Total count:<br>82108<br>T: 779 (1%,<br>645+, 134-)<br>**G: 67238<br>(82%, 54165+,<br>13073-)**<br>C: 292 (0%,	Total count:<br>84394<br>T: 229 (0%,<br>217+, 12-)<br>**G: 69636<br>(83%, 55067+,<br>14569-)**<br>C: 229 (0%,	Total count:<br>88102<br>T: 141 (0%,<br>53+, 88-)<br>G: 1960 (2%,<br>357+, 1603-)<br>C: 214 (0%,<br>17+, 197-)	Total count:<br>83233<br>T: 1128 (1%,<br>575+, 553-)<br>G: 204 (0%,<br>146+, 58-)<br>**C: 75442<br>(91%, 29762+,**	Total count:<br>85379<br>T: 403 (0%,<br>166+, 237-)<br>**G: 79492<br>(93%, 32261+,<br>47231-)**<br>C: 217 (0%,	Total count:<br>88083<br>T: 1701 (2%,<br>965+, 736-)<br>G: 60 (0%,<br>10+, 50-)<br>**C: 78897<br>(90%, 17666+,**	Total count:<br>87914<br>**T: 79176<br>(90%, 18242+,<br>60934-)**<br>G: 128 (0%,<br>31+, 97-)<br>C: 1442 (2%,	*01/null

	522+, 262-)<br>A: 1076 (1%, 438+, 638-)<br>N : 8511 (11%, 5928+, 2583-)<br>---------------<br>DEL: 9002<br>INS: 1753	**(71%, 43844+, 13036-)**<br>A: 370 (0%, 310+, 60-)<br>N : 9207 (11%, 6343+, 2864-)<br>---------------<br>DEL: 2912<br>INS: 2026	**A: 71247 (86%, 58026+, 13221-)**<br>N : 9291 (11%, 6513+, 2778-)<br>---------------<br>DEL: 1036<br>INS: 1370	**14041-)**<br>A: 382 (0%, 366+, 16-)<br>N : 9396 (11%, 6498+, 2898-)<br>---------------<br>DEL: 1741<br>INS: 347	253+, 39-)<br>A: 4370 (5%, 3360+, 1010-)<br>N : 9429 (11%, 6497+, 2932-)<br>---------------<br>DEL: 1548<br>INS: 2928	141+, 88-)<br>A: 4578 (5%, 2978+, 1600-)<br>N : 9722 (12%, 6517+, 3205-)<br>---------------<br>DEL: 877<br>INS: 1872	**A: 79221 (90%, 32867+,** 46354-)<br>N : 6566 (7%, 2592+, 3974-)<br>---------------<br>DEL: 1616<br>INS: 1921	**45680-)**<br>A: 222 (0%, 177+, 45-)<br>N : 6237 (7%, 2206+, 4031-)<br>---------------<br>DEL: 6504<br>INS: 1709	140+, 77-)<br>A: 893 (1%, 224+, 669-)<br>N : 4374 (5%, 1834+, 2540-)<br>---------------<br>DEL: 4436<br>INS: 270	**61231-)**<br>A: 195 (0%, 4+, 191-)<br>N : 7230 (8%, 1567+, 5663-)<br>---------------<br>DEL: 2160<br>INS: 345	588+, 854-)<br>A: 121 (0%, 58+, 63-)<br>N : 7047 (8%, 1556+, 5491-)<br>---------------<br>DEL: 2108<br>INS: 4652	
	chr1:161.629.989	chr1:161.629.983	chr1:161.629.903	chr1:161.629.864	chr1:161.629.853	chr1:161.629.781	chr1:161.626.282	chr1:161.626.250	chr1:161.626.196	chr1:161.624.609	chr1:161.624.517	
BRI_009	Total count: 86156<br>T: 1773 (2%, 1495+, 278-)<br>**G: 25711 (30%, 21074+, 4637-)**<br>**C: 54910 (64%, 42895+, 12015-)**<br>A: 930 (1%, 616+, 314-)<br>N : 2832 (3%, 1304+, 1528-)<br>---------------<br>DEL: 6339<br>INS: 1816	Total count: 88484<br>**T: 59625 (67%, 48412+, 11213-)**<br>G: 1414 (2%, 1010+, 404-)<br>**C: 23494 (27%, 17438+, 6056-)**<br>A: 883 (1%, 301+, 582-)<br>N : 3068 (3%, 1356+, 1712-)<br>---------------<br>DEL: 3772<br>INS: 1526	Total count: 89650<br>T: 381 (0%, 367+, 14-)<br>**G: 53807 (60%, 43532+, 10275-)**<br>C: 208 (0%, 150+, 58-)<br>**A: 31974 (36%, 23875+, 8099-)**<br>N : 3280 (4%, 1514+, 1766-)<br>---------------<br>DEL: 3366<br>INS: 1801	Total count: 90729<br>T: 822 (1%, 624+, 198-)<br>G: 308 (0%, 152+, 156-)<br>**C: 58059 (64%, 44761+, 13298-)**<br>**A: 28229 (31%, 21938+, 6291-)**<br>N : 3311 (4%, 1519+, 1792-)<br>---------------<br>DEL: 2277<br>INS: 707	Total count: 91979<br>T: 537 (1%, 400+, 137-)<br>**G: 27304 (30%, 20764+, 6540-)**<br>C: 469 (1%, 410+, 59-)<br>**A: 60301 (66%, 45956+, 14345-)**<br>N : 3368 (4%, 1543+, 1825-)<br>---------------<br>DEL: 841<br>INS: 1392	Total count: 92386<br>T: 493 (1%, 483+, 10-)<br>G: 874 (1%, 591+, 283-)<br>C: 476 (1%, 311+, 165-)<br>**A: 87048 (94%, 64328+, 22720-)**<br>N : 3495 (4%, 1568+, 1927-)<br>---------------<br>DEL: 2421<br>INS: 614	Total count: 96091<br>T: 187 (0%, 48+, 139-)<br>G: 1687 (2%, 113+, 1574-)<br>C: 233 (0%, 17+, 216-)<br>**A: 93659 (97%, 36670+, 56989-)**<br>N : 325 (0%, 220+, 105-)<br>---------------<br>DEL: 1308<br>INS: 1926	Total count: 90388<br>T: 704 (1%, 434+, 270-)<br>G: 264 (0%, 201+, 63-)<br>**C: 88899 (98%, 33089+, 55810-)**<br>A: 209 (0%, 156+, 53-)<br>N : 312 (0%, 213+, 99-)<br>---------------<br>DEL: 6574<br>INS: 1732	Total count: 95633<br>T: 97 (0%, 26+, 71-)<br>**G: 93926 (98%, 35392+, 58534-)**<br>C: 259 (0%, 161+, 98-)<br>A: 1039 (1%, 238+, 801-)<br>N : 312 (0%, 201+, 111-)<br>---------------<br>DEL: 1643<br>INS: 325	Total count: 91787<br>T: 410 (0%, 362+, 48-)<br>G: 55 (0%, 11+, 44-)<br>**C: 90615 (99%, 18895+, 71720-)**<br>A: 213 (0%, 5+, 208-)<br>N : 494 (1%, 54+, 440-)<br>---------------<br>DEL: 2047<br>INS: 359	Total count: 91518<br>**T: 90491 (99%, 19308+, 71183-)**<br>G: 171 (0%, 28+, 143-)<br>C: 282 (0%, 52+, 230-)<br>A: 147 (0%, 79+, 68-)<br>N : 427 (0%, 52+, 375-)<br>---------------<br>DEL: 1985<br>INS: 4731	**02/*03/*04*
	chr1:161.629.989	chr1:161.629.983	chr1:161.629.903	chr1:161.629.864	chr1:161.629.853	chr1:161.629.781	chr1:161.626.282	chr1:161.626.250	chr1:161.626.196	chr1:161.624.609	chr1:161.624.517	
BRI_010	Total count: 43314<br>T: 882 (2%, 745+, 137-)<br>**G: 14824 (34%, 12437+, 2387-)**<br>**C: 26399 (61%, 20399+, 6000-)**<br>A: 544 (1%, 361+, 183-)<br>N : 665 (2%, 292+, 373-)<br>---------------<br>DEL: 3278<br>INS: 914	Total count: 44461<br>**T: 29232 (66%, 23719+, 5513-)**<br>G: 681 (2%, 480+, 201-)<br>**C: 13336 (30%, 10259+, 3077-)**<br>A: 488 (1%, 177+, 311-)<br>N : 724 (2%, 304+, 420-)<br>---------------<br>DEL: 1982<br>INS: 780	Total count: 44720<br>T: 216 (0%, 213+, 3-)<br>**G: 25569 (57%, 20619+, 4950-)**<br>C: 142 (0%, 98+, 44-)<br>**A: 18004 (40%, 14021+, 3983-)**<br>N : 789 (2%, 359+, 430-)<br>---------------<br>DEL: 1707<br>INS: 887	Total count: 45322<br>T: 427 (1%, 341+, 86-)<br>G: 131 (0%, 64+, 67-)<br>**C: 33252 (73%, 25964+, 7288-)**<br>**A: 10726 (24%, 8376+, 2350-)**<br>N : 786 (2%, 362+, 424-)<br>---------------<br>DEL: 1099<br>INS: 285	Total count: 45874<br>T: 297 (1%, 221+, 76-)<br>**G: 15751 (34%, 12534+, 3217-)**<br>C: 233 (1%, 202+, 31-)<br>**A: 28791 (63%, 21876+, 6915-)**<br>N : 802 (2%, 356+, 446-)<br>---------------<br>DEL: 430<br>INS: 760	Total count: 46362<br>T: 241 (1%, 233+, 8-)<br>G: 6569 (14%, 5016+, 1553-)<br>C: 243 (1%, 163+, 80-)<br>**A: 38430 (83%, 28378+,** 10052-)<br>N : 879 (2%, 352+, 527-)<br>---------------<br>DEL: 1221<br>INS: 469	Total count: 47830<br>T: 85 (0%, 25+, 60-)<br>G: 960 (2%, 65+, 895-)<br>C: 105 (0%, 5+, 100-)<br>**A: 46488 (97%, 16757+, 29731-)**<br>N : 192 (0%, 132+, 60-)<br>---------------<br>DEL: 700<br>INS: 1035	Total count: 45197<br>T: 335 (1%, 200+, 135-)<br>G: 118 (0%, 74+, 44-)<br>**C: 44447 (98%, 15127+, 29320-)**<br>A: 113 (0%, 82+, 31-)<br>N : 184 (0%, 123+, 61-)<br>---------------<br>DEL: 3307<br>INS: 862	Total count: 47613<br>T: 50 (0%, 9+, 41-)<br>**G: 46731 (98%, 16264+, 30467-)**<br>C: 128 (0%, 80+, 48-)<br>A: 516 (1%, 113+, 403-)<br>N : 188 (0%, 126+, 62-)<br>---------------<br>DEL: 898<br>INS: 175	Total count: 47859<br>T: 202 (0%, 183+, 19-)<br>G: 28 (0%, 3+, 25-)<br>**C: 47167 (99%, 9604+, 37563-)**<br>A: 99 (0%, 3+, 96-)<br>N : 363 (1%, 49+, 314-)<br>---------------<br>DEL: 1043<br>INS: 194	Total count: 47854<br>**T: 47241 (99%, 9949+, 37292-)**<br>G: 72 (0%, 16+, 56-)<br>C: 144 (0%, 26+, 118-)<br>A: 89 (0%, 49+, 40-)<br>N : 308 (1%, 47+, 261-)<br>---------------<br>DEL: 1059<br>INS: 2501	**02/*03/*04*

Table S5 Primer design in-house TaqMan PCR

**Nucleotide**		**Sequence**	**Gen. Loc.**
c.114	Fwd	CCTGGCACTTCAGAGTCACA	1:161548595-161548614 (FCGR3A)<br>1:161629952-161629971 (FCGR3B)
	Rev	CCTGGAGCCTCAATGGTACAG	1:161548633-161548653 (FCGR3A)<br>1:161629990-161630010 (FCGR3B)
c.194	Fwd	CCTGAGGACAATTCCACACAGT	1:161548562-161548583 (FCGR3A)<br>1:161629919-161629940 (FCGR3B)
	Rev	CGAGGCCTGGCTTGAGA	1:161548524-161548540 (FCGR3A)<br>1:161629881-161629897 (FCGR3B)
c.233	Fwd	GCACCTGTACTCTCCACTGT	1:161548473-161548492 (FCGR3A)<br>1:161629830-161629849 (FCGR3B)
	Rev	AGCCAGGCCTCGAGCTA	1:161548519-161548535 (FCGR3A)<br>1:161629876-161629892 (FCGR3B)
c.244	Fwd	GGCCTCGAGCTACTTCATTGAC	1:161548509-161548530 (FCGR3A)<br>1:161629866-161629887 (FCGR3B)
	Rev	GTTTGTCTGGCACCTGTACTCT	1:161548464-161548485 (FCGR3A)<br>1:161629821-161629842 (FCGR3B)
c.316	Fwd	AACCTCTCCACCCTCAGTGA	1:161548447-161548466 (FCGR3A)<br>1:161629804-161629823 (FCGR3B)
	Rev	GGTGATTTTCCTCTTCCCCTTCATC	1:161548389-161548413 (FCGR3A)<br>1:161629746-161629770 (FCGR3B)

Table S6 Primer design long-read sequencing

Region	Sequence	Gen. Loc.
Fwd	GCTCTTTCCTTCCTATTCCTGT	1:161631285-161631306 (FCGR3B)
Rev	TCCCCCTTAGCCCAATTTTCTC	1:161623044-161623065 (FCGR3B)
